# Therapeutic potential of *Lachnospiraceae* strains in irritable bowel syndrome via differential gut-brain pathways

**DOI:** 10.20517/mrr.2026.08

**Published:** 2026-05-29

**Authors:** Wanyu Yang, Shuigen Bian, Huizi Tan

**Affiliations:** State Key Laboratory of Food Science and Resources, China-Canada Joint Laboratory of Food Science and Technology (Nanchang), Nanchang University, Nanchang 330047, Jiangxi, China.

**Keywords:** Irritable bowel syndrome, *Blautia* *wexlerae*, *Roseburia faecis*, *Dorea* *longicatena*, *Coprococcus* *eutactus*, gut-brain axis

## Abstract

**Background:** Irritable bowel syndrome (IBS) is a highly prevalent disorder that significantly impairs quality of life, yet effective treatments remain limited. While prior work from our laboratory linked the *Lachnospiraceae* family to IBS severity, conflicting sequencing data necessitated a deeper investigation into this taxonomic group.

**Methods:** We evaluated the therapeutic potential of four representative *Lachnospiraceae* strains in IBS mice with standardized gut microbiota baseline (SynCom-23). Core symptoms were assessed via body weight, fecal status, and visceral hypersensitivity. Intestinal inflammation, oxidative stress, and barrier function were evaluated by enzyme-linked immunosorbent assay (ELISA) and quantitative real-time polymerase chain reaction (RT-qPCR), and gut microbiota and metabolites were analyzed by 16S rRNA sequencing, gas chromatography-flame ionization detector (GC-FID), and liquid chromatography-mass spectrometry (LC-MS), together with brain neurotransmitter systems.

**Results:** We found that all four strains effectively alleviated IBS symptoms, reducing weight loss (*P* < 0.001) and visceral hypersensitivity markers calcitonin gene-related peptide (CGRP) and transient receptor potential vanilloid 1 (TRPV1) (*P* < 0.0001), potentially through distinct pathways: *Blautia wexlerae* MW-022 produces substantial acetic acid, stimulates *Lactobacillaceae* proliferation, and targets the γ-aminobutyric acid/glutamate (GABA/Glu) system. *Roseburia faecis* MW-024 produces butyrate, affecting the GABA/Glu system. *Dorea longicatena* MW-023 enhances the serotonin (5-HT) system at synthesis and reception levels; and *Coprococcus eutactus* DSM107541 modulates the 5-HT system via the “clearance-reception” mode. Combined strains achieved more comprehensive gut micro-ecology restoration.

**Conclusion:** This study systematically elucidates the heterogeneity in the mechanisms by which *Lachnospiraceae* strains treat IBS at the strain level. Different strains achieve precise regulation through their specific gut-brain axes. These findings offer strain-level insights and a foundation for developing *Lachnospiraceae*-based microbial therapies.

## INTRODUCTION

Global epidemiological studies indicate that irritable bowel syndrome (IBS) affects 5%-10% of the population worldwide^[1]^. The prevalence rates range from 4-10% in Asian countries to as high as 9%-22% in Western nations^[2]^. Characterized by persistent or recurrent abdominal pain and altered bowel habits, IBS significantly impairs patients’ quality of life^[3]^. Its pathophysiology is multifactorial, involving low-grade inflammation^[4]^, gut-brain axis dysfunction^[5]^, psychological comorbidities such as anxiety and depression^[6,7]^, altered gastrointestinal motility^[8]^, visceral hypersensitivity, and gut microbiota dysbiosis^[9,10]^. Given the disease’s heterogeneity and complexity, current treatment options are often suboptimal. Therefore, elucidating the specific roles of key gut microbial members in IBS pathogenesis and their potential gut-brain axis mechanisms has become a focused research direction. This line of investigation holds promise for developing targeted microbial therapies.

A previous meta-analysis from our laboratory investigating diet and IBS revealed a strong correlation between gut microbiota composition and IBS symptoms^[11]^. Subsequent analysis suggested that *Lachnospiraceae* family is significantly involved in the disease process^[12]^. However, sequencing data presented conflicting evidence regarding the effects of this family, prompting our specific focus on this taxonomic group. This study aims to systematically explore the therapeutic effects of representative *Lachnospiraceae* strains on IBS and their underlying gut-brain axis mechanisms. By doing so, we sought to clarify the functional heterogeneity among phylogenetically related strains. Literature indicates that *Roseburia* abundance is significantly reduced in IBS patients^[13]^. Notably, *Roseburia faecis* (*R. faecis*) has been shown to exert anti-inflammatory, gut-brain axis modulatory, and intestinal barrier-protective effects, alleviating symptoms in a rat IBS model^[14]^. Similarly, reduced *Blautia* abundance has been reported in IBS patients^[15]^. *Blautia wexlerae* (*B. wexlerae*), in particular, is considered a promising probiotic candidate due to its ability to produce short chain fatty acids (SCFAs), regulate bile acids (BAs) mmetabolism, and maintain barrier integrity^[16]^. Regarding *Coprococcus*, a human study by Chen *et al.*^[17]^. hypothesized that its reduced abundance disrupts host-microbial metabolic interactions, leading to immune dysregulation. Another investigation further linked *Coprococcus eutactus* (*C. eutactus*) levels to IBS symptom severity^[18]^. *Dorea* also presents a complex profile within the *Lachnospiraceae* family. Although often reduced in IBS patients^[19]^, some clinical studies suggest that decreased *Dorea* levels correlate with effective clinical improvement^[20]^. This duality may be explained by *Dorea longicatena* (*D. longicatena*), which generates beneficial short-chain fatty acids but also produces gas, resulting in context-dependent effects on the host^[21]^.

In light of these findings, we selected four representative *Lachnospiraceae* strains (*B. wexlerae* MW-022, *R. faecis* MW-024, *D. longicatena* MW-023, and *C. eutactus* DSM107541) and evaluated their therapeutic effects in an IBS mouse model under a standardized gut microbiota background (SynCom-23). Key endpoints included core IBS symptoms, intestinal inflammation, oxidative stress, barrier integrity, gut microbiota and metabolites, and brain neurotransmitter systems.

## METHODS

### Microbial strains and cultivation

The strains *Blautia wexlerae* MW-022, *Dorea* *longicatena* MW-023, and *Roseburia* *faecis* MW-024 were originally isolated from fecal samples of healthy adults and deposited at the State Key Laboratory of Food Science and Resources. *Coprococcus eutactus *DSM107541 was purchased from DSMZ-German Collection of Microorganisms and Cell Cultures GmbH, Germany. All microorganisms were cultured in yeast extract-casitone-fatty acids (YCFA) medium (Hopebio, China) under anaerobic conditions of 5% H_2_, 5% CO_2_, and 90% N_2_ at 37 °C^[22]^.

### Animal experiments

A total of 42 six-week-old male C57BL/6J mice with an initial body weight of approximately 21 g were obtained from Nanjing GemPharmatech. They were maintained under specific pathogen-free (SPF) conditions with a 12-h light/dark cycle and provided with ad libitum access to standard chow and water. After seven days of acclimation, the mice were randomized into seven groups (*n* = 6 per group): a normal control group (B), a model group (M), and five intervention groups.

All mice except those in the B group underwent gut microbiota depletion via a 7-day oral antibiotic cocktail (ampicillin 100 mg/kg, metronidazole 100 mg/kg, vancomycin 50 mg/kg, and neomycin 100 mg/kg)^[23]^. Beginning on day 3 of antibiotic treatment, IBS was induced by daily oral gavage of senna leaf decoction (0.5 g/mL, 10 mL/kg BW) combined with restraint stress for 15 days^[24-26]^

Following a one-day washout of antibiotics, the gut microbiota of these mice was reconstructed by oral gavage of a synthetic community (SynCom-23) for 3 consecutive days^[27-29]^. SynCom-23 was composed of 23 strains detailed in Supplementary Table 1. Individual strains were grown anaerobically in YCFA medium at 37 °C and adjusted to the concentrations listed in Supplementary Table 1. Equal volumes of each culture were then combined to produce a final suspension at 2.1 × 10^9^ colony forming unit (CFU)/mL; 0.2 mL of this suspension was administered to each mouse.

Immediately following the SynCom-23 reconstruction, the 10-day bacterial intervention phase began. Mice in the R.f, D.l, B.w, and C.e groups received oral gavage of 10^8^ CFU live cells of *R. faecis* MW-024, *D. longicatena* MW-023, *B. wexlerae* MW-022, and *C. eutactus* DSM107541, respectively, each suspended in 0.2 mL phosphate-buffered saline (PBS) (pH 7.2). The mix group was given a mixture of the four bacteria cells (10^8^ CFU in total) by gavage. Meanwhile, the mice in the B and M groups received equivalent volumes of PBS as a vehicle control.

Throughout the experiment, the body weight and fecal status of the mice were monitored daily. The time to first loose stool was defined as the interval from the end of senna leaf gavage to the first observation of loose stool (unformed, watery feces with perianal soiling). This parameter was recorded independently by two blinded researchers. Fecal consistency was assessed semi-quantitatively using the Bristol Stool Form Scale^[30]^. Upon completion of the study, the mice were anesthetized and sacrificed by cervical dislocation. Serum samples, fecal samples, colon tissues including contents, and prefrontal cortex were carefully collected and appropriately preserved for subsequent analysis.

All animal experiments in this study complied with the National Institutes of Health Guidelines for the Care and Use of Laboratory Animals. They were approved by the Experimental Animal Care and Use Committee of Nanchang University (approval No. NCULAE-20250916003).

### Behavioral tests

**The Y-maze test^[31]^** was employed to assess spatial working memory, as originally described by Dellu et al. The apparatus consisted of a custom-built maze with three identical arms (50 cm × 18 cm × 35 cm) joined at a 120° angle. Distinct geometric shapes were affixed to the inner surface of each arm for visual differentiation. For each trial, a mouse was initially placed in the central junction, facing a randomly selected arm, and allowed to explore freely for 5 min. Its behavior was recorded by a video tracking system. The following parameters were documented: Entries into an arm were counted whenever all four paws crossed into the arm (total arm entries). Spontaneous alternation was recorded when a mouse entered three different arms consecutively. The percentage of spontaneous alternation was calculated as [Number of Alternations / (Total Arm Entries - 2)] × 100%. After each trial, the maze was wiped down with 75% ethanol to remove any odor cues.

**The open field test^[32]^** was conducted to evaluate general locomotor activity and anxiety-like behavior. Prior to testing, all animals were acclimatized to the behavioral testing room for 2 h under quiet, sound-attenuated conditions. Each mouse was gently placed in the center of a white, square open-field arena (40 cm × 40 cm × 60 cm) and allowed to explore freely for 5 min. An overhead camera, fixed 1.5 m above the arena, recorded the movements. Video tracking software was used to quantify total distance traveled as a measure of locomotor activity. Anxiety-like behavior was inferred from the time spent and the number of entries into the central zone. Reduced activity in this zone was taken as an indicator of higher anxiety. The arena was cleaned with 75% ethanol between each trial. Each subsequent test began only after the ethanol had fully evaporated.

### Histopathological analysis

Colon tissue samples were fixed in 4% paraformaldehyde, embedded in paraffin, and sectioned at a thickness of 4 μm. Sections were then stained with hematoxylin and eosin (H&E)^[33]^. Whole-slide imaging was performed using a digital pathology scanning system (Leica Aperio LV1).

### Gut microbiota analysis

Fresh fecal pellets were collected from each animal, placed in sterile tubes, and stored at -80 °C. Total genomic DNA was isolated with the FastPure Stool DNA Isolation Kit (MJYH, Shanghai, China) following the manufacturer’s protocol. DNA concentration and purity were measured on a NanoDrop 2000 spectrophotometer (Thermo Fisher Scientific, USA). The V3-V4 hypervariable region of the 16S rRNA gene was amplified using the barcoded primer pair 338F/806R^[34]^ on an ABI GeneAmp® 9700 thermal cycler. The amplicons were cleaned up with a DNA Gel Extraction Kit (PCR Clean-Up Kit, YuHua, China) and quantified on a Qubit 4.0 fluorometer (Thermo Fisher Scientific, USA). Equimolar quantities of the purified products were then combined and sequenced in paired-end mode on an Illumina NextSeq 2000 platform (Illumina, San Diego, USA). Sequencing was performed by Majorbio Bio-Pharm Technology Co. Ltd. (Shanghai, China).

Raw sequencing data were demultiplexed, quality-filtered with fastp (version 0.19.6)^[35]^, and merged with FLASH (version 1.2.11)^[36]^. Subsequently, the DADA2^[37] ^plugin within the QIIME2 (version 2020.2)^[38]^ pipeline was employed to denoise the sequences and resolve them into amplicon sequence variants (ASVs). To standardize for sequencing depth, the ASV table was rarefied to 20,000 sequences per sample for downstream alpha and beta diversity analyses. This rarefaction depth achieved an average Good's coverage of 97.90%. Taxonomic classification of ASVs was performed using the Naive Bayes consensus taxonomy classifier in QIIME2 against the SILVA 16S rRNA database (version 138).

Bioinformatic and statistical data analyses were conducted on the Majorbio Cloud Platform. Alpha diversity indices (Chao1 and Sobs) were calculated using mothur^[39]^, and differences between groups were assessed using the Wilcoxon rank-sum test. For beta diversity, Bray-Curtis dissimilarities were calculated and visualized using Principal Coordinate Analysis (PCoA). Permutational multivariate analysis of variance (PERMANOVA) was applied to test for significant differences in microbial community structure across groups. Linear discriminant analysis effect size (LEfSe) was employed to identify bacterial taxa with significantly different abundances between groups. Taxa detected from phylum to genus level were retained with an linear discriminant analysis (LDA) score > 2 and *P* < 0.05. Distance-based redundancy analysis (db-RDA) was used to investigate the influence of clinical indicators on gut bacterial community structure. Subsequently, linear regression analysis was applied to assess the impact of these key clinical indicators on alpha diversity indices. Finally, a Spearman correlation network^[41]^ was constructed to visualize microbial co-occurrence patterns (|*r*| > 0.6, *P* < 0.05).

### Gene expression analysis

Total RNA was reverse transcribed into cDNA using the PrimeScript RT Reagent Kit (Takara). Subsequently, quantitative real-time PCR (RT-qPCR) was carried out using TB Green Premix Ex Taq II (Takara). The reactions were run on a QuantStudio 7 Flex Real-Time PCR System (Thermo Fisher Scientific, Waltham, MA, USA). This assay was used to quantify the mRNA expression of genes related to visceral hypersensitivity, tight junction integrity, and neurotransmitter systems. Primer sequences are listed in Supplementary Table 2. β-actin served as the endogenous reference gene for normalization, and relative gene expression levels were calculated using the 2^-ΔΔCt^ method^[33]^.

### Enzyme-linked immunosorbent assay

Distal colon specimens (30 mg) were homogenized in ice-cold PBS, and the homogenates were centrifuged at 10,000 × g for 10 min at 4 °C^[42]^. The supernatants were then harvested for subsequent assays. Levels of myeloperoxidase (MPO), malondialdehyde (MDA), superoxide dismutase (SOD), interleukin-10 (IL-10), tumor necrosis factor-α (TNF-α), and interleukin-6 (IL-6) in the colon were determined using commercial enzyme-linked immunosorbent assay (ELISA) kits (Fcmacs, Nanjing, China) according to the manufacturer’s instructions. All measurements were normalized to the total protein content of each sample, as quantified with a BCA protein assay kit (Beyotime, Shanghai, China).

### Analysis of metabolites in gut contents and bacterial culture

#### SCFAs

For bacteria fermentation samples cultured for 48 h or 24 h, 1 mL of broth was centrifuged at 13,000 rpm for 5 min. Cecal contents (100 mg) from mice were homogenized in 1 mL of physiological saline at 70 Hz for 1 min. The homogenate was vortexed for 3 min and centrifuged at 13,000 rpm for 5 min^[43]^.

The supernatant was filtered through a 0.22 μm aqueous-phase membrane and mixed with 0.2 mL of 10% (v/v) sulfuric acid. The mixture was then vortexed for 1 min. Subsequently, 10 μL of 10 mM internal standard (2-Ethylbutyric acid) and 0.4 mL of anhydrous ethyl ether were added. After vortexing and incubation for 2 min, the samples were centrifuged at 13,000 rpm for 2 min. The organic layer was passed through a 0.22 μm organic membrane into a vial. Analysis was performed on an Agilent 7890B GC system equipped with an HP-FFAP capillary column and a flame ionization detector (FID)^[44]^. A mixed external standard was used as a reference^[45]^.

High-purity nitrogen served as the carrier gas (2 mL/min), with high-purity hydrogen (30 mL/min) and air (400 mL/min) used as the fuel and makeup gases, respectively. The injector was maintained at 240°C, employing a 5:1 split ratio and a septum purge flow of 3 mL/min. The injection volume was set to 2 μL. The oven temperature program was configured as follows: initial temperature of 80 °C, increased to 140 °C at a ramp rate of 7.5 °C/min, then raised to 200 °C at 15 °C/min, and held at 200 °C for 3 min. The detector temperature was kept constant at 300 °C.

#### Bile acids and neurotransmitters

After the mice were sacrificed, the ileal segment was immediately dissected longitudinally. The adherent content on the intestinal wall was gently scraped off using a sterile glass slide, quickly collected in a pre-cooled sterile cryotube, and snap-frozen in liquid nitrogen to minimize metabolite degradation. Ileal contents (25 mg wet weight) were homogenized with 700 μL of pre-cooled extraction solution (MeOH:H_2_O = 4:1, containing internal standards) using a tissue homogenizer (40 Hz, 4 min). This was followed by ultrasonication in an ice-water bath. The homogenization and sonication cycle was repeated twice. The extracts were incubated at -40 °C for 1 h, centrifuged (4 °C, 12,000 rpm, 15 min), and the supernatants were collected for analysis. Quality control (QC) samples were prepared by pooling equal volumes of all samples^[46]^.

Chromatographic separation was performed on an Agilent 1290 UHPLC system equipped with an ACQUITY BEH C18 column. The mobile phase consisted of ultrapure water (A) and methanol-water (95:5, v/v) (B). Mass spectrometry detection was conducted on a SCIEX 6500 QTRAP+ system with an ESI source in MRM mode. Source parameters were: curtain gas, 35 psi; source gases, 50 psi; temperature, 450℃; and ion spray voltage, ± 4,500/5,000 V. Raw data were acquired using SCIEX Analyst software (v1.7.32), and quantification was performed using BIOTREE Bio Bud (v2.0.3). Chromatographic performance was monitored via total ion chromatogram (TIC).

### Statistical analysis

All statistical analyses were performed using GraphPad Prism (version 10.1.0) and SPSS (version 26.0). Data are presented as mean ± standard error of the mean (SEM). For two-group comparisons, Student’s *t*-test was used. For multiple-group comparisons, one-way analysis of variance (ANOVA) followed by Tukey's post hoc test was applied to control the family-wise error rate. For correlation analyses involving multiple variables, Spearman's rank correlation coefficients were computed using the R packages psych, pheatmap, and reshape2. Raw *P*-values from these analyses were corrected using the Benjamini-Hochberg false discovery rate (FDR) procedure, with statistical significance set at adjusted *q* < 0.05. A two-tailed *P*-value (or adjusted *q*-value) of less than 0.05 was considered statistically significant for all tests unless otherwise stated.

## RESULTS

### *Lachnospiraceae* strains ameliorate IBS symptoms in mice

**Figure 1:**
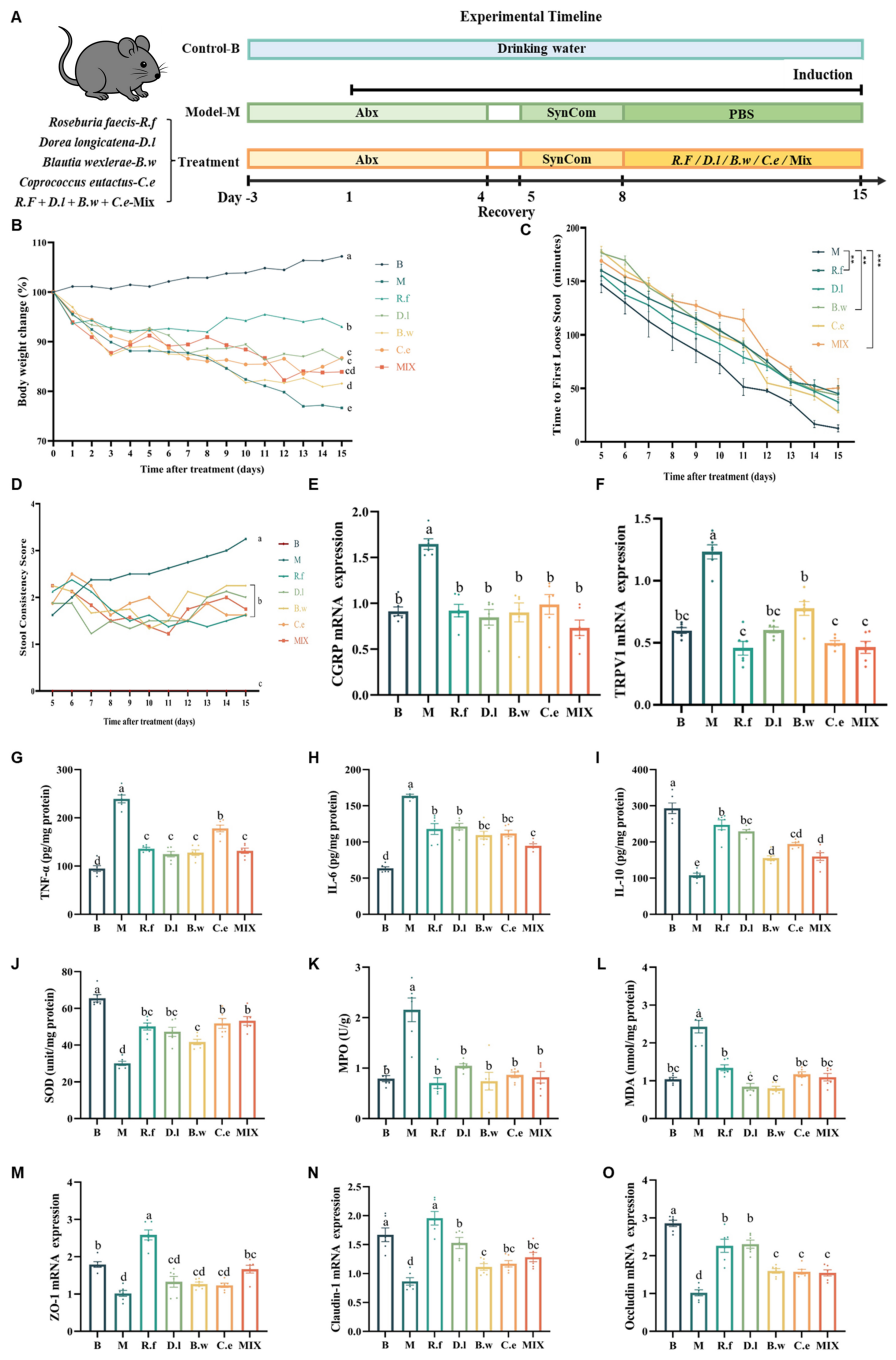
*Lachnospiraceae* strains ameliorate IBS symptoms in mice. (A) Experiment design; (B) Body weight changes; (C) Time to first loose stool; (D) Fecal consistency scores; (E and F) Colonic expression levels of visceral hypersensitivity-related factors CGRP and TRPV1. Different letters indicate statistical difference among groups; (G-I) Colonic expression levels of cytokines TNF-α, IL-6, and IL-10; (J-L) Colonic expression levels of oxidative stress markers SOD, MPO, and MDA; (M-O) Colonic expression levels of tight junction proteins ZO-1, Claudin-1, and Occludin. Data are presented as mean ± SEM. Different letters indicate statistically significant differences among groups (one-way ANOVA followed by Tukey HSD post hoc test, *P *< 0.05). Asterisks denote: **P < 0.01, ***P < 0.001. PBS: Phosphate-buffered saline; TNF-α: tumor necrosis factor-α; IL-6: interleukin-6; IL-10: interleukin-10; IBS: irritable bowel syndrome; CGRP: calcitonin gene-related peptide; TRPV1: transient receptor potential vanilloid 1; SOD: superoxide dismutase; MPO: myeloperoxidase; MDA: malondialdehyde; ZO-1: zonula occludens-1; SEM: standard error of the mean; ANOVA: analysis of variance; HSD: honest significant difference.

We established a standardized IBS model by first depleting the endogenous gut microbiota of mice with an antibiotic cocktail, followed by colonization with SynCom-23 [Figure 1A]. Following model establishment, mice exhibited significant weight loss; however, this effect was substantially alleviated by intervention with the four selected *Lachnospiraceae* strains [Figure 1B]. Regarding diarrheal symptoms, treatments with *B. wexlerae* MW-022, *R. faecis* MW-024, or the four-strain combination significantly delayed the onset of diarrhea. This was indicated by the prolonged time to first loose stool [Figure 1C]. Notably, all interventions improved stool consistency [Figure 1D]. At the molecular level, all treatments significantly modulated the colonic expression of visceral hypersensitivity markers, calcitonin gene-related peptide (CGRP) and transient receptor potential vanilloid 1 (TRPV1) [Figure 1E and F]. Consistent with the core pathology of IBS, which is characterized by the absence of overt organic lesions^[5]^, histological analysis revealed no significant structural damage to the colonic mucosa in any group [Supplementary Figure 1]. Collectively, these data confirmed the successful establishment of an IBS mouse model and demonstrated that the selected *Lachnospiraceae* strains effectively ameliorated core IBS phenotypes and regulated visceral sensitivity.

### *Lachnospiraceae* strains improve intestinal homeostasis in IBS mice

All interventions significantly reduced the levels of pro-inflammatory cytokines TNF-α and IL-6, while increasing anti-inflammatory cytokine IL-10 [Figure 1G-I]. Furthermore, all *Lachnospiraceae* strains effectively modulated key oxidative stress indicators: compared with the M group, all interventions elevated SOD content, albeit not to normal levels, and reduced MPO activity and MDA content to near-normal levels [Figure 1J-L], indicating a role in mitigating intestinal low-grade inflammation and oxidative damage. To assess intestinal barrier integrity, we measured the expression of tight junction proteins. Among all strains, *R. faecis* MW-024 significantly upregulated the expression of ZO-1, Claudin-1, and Occludin. The other three single-strain interventions also effectively upregulated the expression of Claudin-1 and Occludin, but showed strain-specific differences in the regulation of zonula occludens-1 (ZO-1): *B. wexlerae* MW-022, *D. longicatena* MW-023, and *C. eutactus* DSM107541 did not significantly alter the expression level of ZO-1 [Figure 1M-O]. Notably, *R. faecis* MW-024 exhibited the most pronounced upregulation effect on these three tight junction proteins, underscoring its potent capacity to enhance the intestinal barrier function.

### *Lachnospiraceae* strains differentially alleviate neurological abnormalities in IBS mice

**Figure 2:**
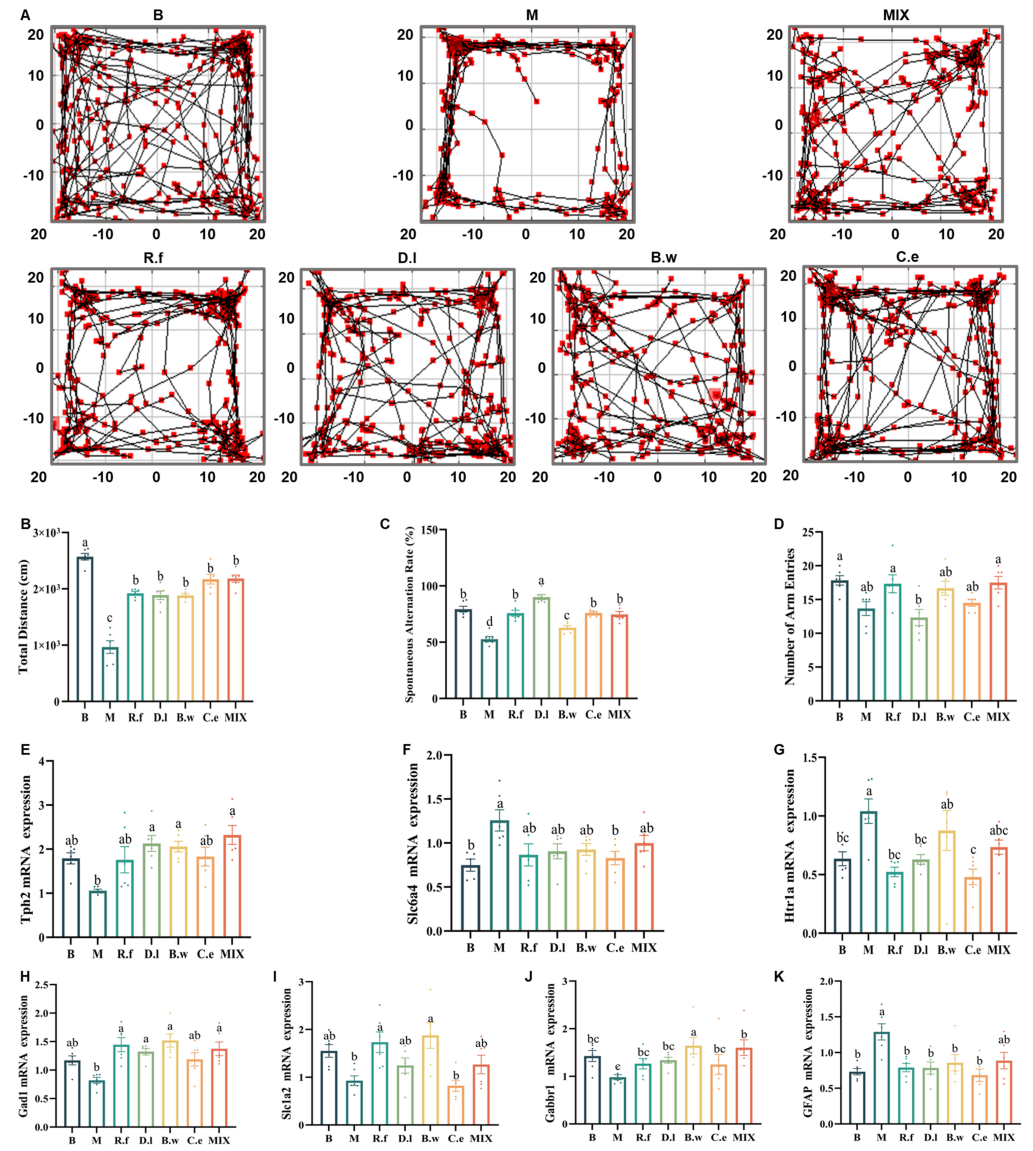
* Lachnospiraceae* strains differentially alleviate neurological abnormalities in IBS mice. (A) Representative movement trajectories of mice in the open field test; (B) Total distance moved in the open field; (C) Spontaneous alternation rate (%) in the Y-maze test; (D) Total number of arm entries in the Y-maze test; (E-G) Expression levels of markers involved in the 5-HT system (*Tph2*, *Slc6a4*, and *Htr1a*) in the prefrontal cortex; (H-K) Expression levels of factors involved in the GABA/Glu system (*Gad1*, *Slc1a2*, and *Gabbr1*) and the astrocytic marker GFAP in the prefrontal cortex. Note: Data are presented as mean ± SEM. Different letters indicate statistically significant differences among groups (one-way ANOVA followed by Tukey HSD post hoc test, *P* < 0.05). GFAP: Glial fibrillary acidic protein; IBS: irritable bowel syndrome; 5-HT: serotonin; SEM: standard error of the mean; ANOVA: analysis of variance; HSD: honest significant difference.

To assess the therapeutic potential of *Lachnospiraceae* strains on IBS-related behavioral abnormalities, we first characterized the deficits in our mouse model using the open field and Y-maze tests. These tests are commonly used to capture anxiety-like behavior and spatial working memory mediated through the gut-brain axis. Compared to the B group, IBS mice exhibited significant anxiety-like behavior and diminished exploratory motivation, evidenced by reduced time in the central zone and decreased total distance traveled in the open field test [Figure 2A and B]. In the Y-maze test, they showed a specific impairment in spatial working memory, indicated by a significantly reduced spontaneous alternation rate, while general locomotion was unaffected [Figure 2C and D].

Following intervention with the *Lachnospiraceae* strains, these anxiety-like behaviors and diminished exploratory motivation were effectively reversed. All treatments also alleviated the cognitive impairment observed in the Y-maze test, though with varying degrees of efficacy. Notably, the D.l group achieved the highest spontaneous alternation rate. The R.f, C.e and Mix groups restored cognitive function to a level equivalent to the B group. While *B. wexlerae* MW-022 also significantly increased the spontaneous alternation rate, its performance remained significantly lower than that of the B group. To elucidate the underlying neural mechanisms, we further examined the expression of key factors in neurotransmitter systems within the prefrontal cortex^[47]^.

IBS mice exhibited significant dysregulation in both the serotonin (5-HT) system^[48]^ [Figure 2E-G] and the GABA/glutamate (GABA/Glu) system^[49]^ [Figure 2H-K]. Each strain exerted distinct, strain-specific neuromodulatory effects on these pathways. Within the 5-HT system, *C. eutactus* DSM107541 reversed the expression of *Slc6a4* and *Htr1a*; *D. longicatena* MW-023 reversed the expression of *Tph2* and *Htr1a*; both reversed two out of the three key indicators (*Tph2*, *Slc6a4* and *Htr1a*) respectively, thus achieving a more prominent effect. In contrast, *R. faecis* MW-024 solely downregulated *Htr1a*, while *B. wexlerae* MW-022 and the mixed strains specifically upregulated *Tph2*. Regarding the GABA/Glu system, *B. wexlerae* MW-022 demonstrated the most potent capacity, significantly regulating all three markers (*Gad1*, *Slc1a2*, and *Gabbr1*). *R. faecis *MW-024 notably regulated two of the three markers (*Gad1* and *Slc1a2*), whereas *D. longicatena* MW-023 solely reversed abnormalities in *Gad1*. The mixed strains upregulated *Gad1* and *Gabbr1*. Additionally, all four strains effectively downregulated the astrocytic marker glial fibrillary acidic protein (GFAP), with the exception of the mixed formulation. 

### *Lachnospiraceae* strains differentially modulate host metabolite profiles in IBS mice

**Figure 3:**
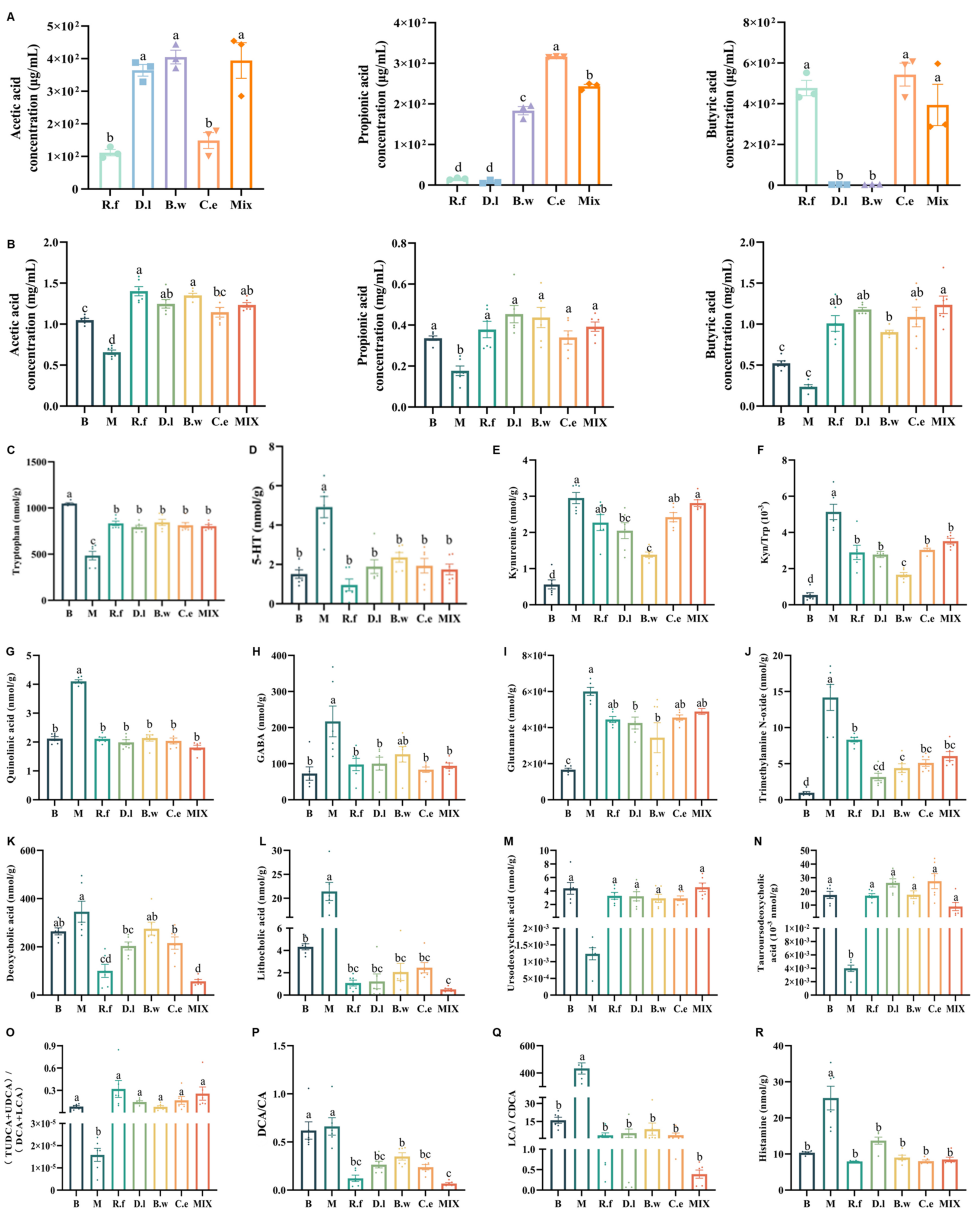
*Lachnospiraceae* strains differentially modulate host metabolite profiles in IBS mice. (A) SCFAs produced by the four *Lachnospiraceae* strains and their mixture; (B) SCFAs in cecal contents; (C-F) Metabolites related to tryptophan-serotonin pathway in ileal contents: 5-HT, Trp, kynurenine (KYN), and the KYN/Trp ratio; (G) Quinolinic acid in ileal contents; (H-J) Metabolites related to GABA/Glu pathway in ileal contents: GABA, glutamate, and trimethylamine N-oxide; (K-N) BAs in ileal contents: DCA, LCA, UDCA, and TUDCA; (O-Q) BAs functional indices: (UDCA+TUDCA)/(LCA+DCA), DCA/CA, and LCA/CDCA ratios. (R) Histamine in ileal contents. Note: Data are presented as mean ± SEM. Different letters indicate statistically significant differences among groups (one-way ANOVA followed by Tukey HSD post hoc test, *P* < 0.05). 5-HT: Serotonin; GABA: γ-aminobutyric acid; Trp: tryptophan; TUDCA: tauroursodeoxycholic acid; UDCA: ursodeoxycholic acid; DCA: deoxycholic acid; LCA: lithocholic acid; CA: cholic acid; CDCA: chenodeoxycholic acid; SCFA: short chain fatty acid; BA: bile acid; SEM: standard error of the mean; ANOVA: analysis of variance; HSD: honest significant difference.

To elucidate the potential impact of *Lachnospiraceae* on host metabolism, we first analyzed the intrinsic metabolic capabilities of the individual strains and their combination. The analysis focused on the production of SCFAs and neuroactive metabolites. Analysis of SCFA production revealed distinct functional specialization among strains [Figure 3A]. *R. faecis* MW-024 and *C. eutactus* DSM107541 were identified as efficient butyrate producers, whereas *B. wexlerae* MW-022 and *D. longicatena* MW-023 primarily generated acetate. Additionally, *B. wexlerae* MW-022 and *C. eutactus* DSM107541 produced modest amounts of propionate. The mixed-strain combination exhibited metabolic complementarity, simultaneously producing multiple SCFAs. Furthermore, *in vitro* cultures confirmed that all four *Lachnospiraceae* strains synthesized GABA at comparable levels [Supplementary Figure 2A]. 

*In vivo* experiments revealed that the four strains significantly increased the colonic concentrations of acetic, propionic, and butyric acid in IBS mice. This effect was observed both when strains were administered individually and in combination [Figure 3B].

At the neuromodulatory level, these interventions partially reversed the neurological abnormalities induced by the IBS-D model, albeit through strain-specific pathways. Analysis of the tryptophan-serotonin pathway revealed that *D. longicatena* MW-023 and *B. wexlerae* MW-022 were the most effective modulators. These strains elevated tryptophan (Trp) levels while reducing serotonin (5-HT) and kynurenine (KYN) concentrations, thereby normalizing the KYN/Trp ratio [Figure 3C-F]. In contrast, *R. faecis* MW-024, *C. eutactus *DSM107541, and the mixed strains did not effectively recover KYN levels. Additionally, all four strains notably reversed the expression of quinolinic acid (QUIN) [Figure 3G]. Regarding the GABA/Glu pathway, all treatments, with the exception of the B.w group, reduced GABA concentrations [Figure 3H]. Conversely, both D.l and B.w groups demonstrated restoration of glutamate levels [Figure 3I]. A common effect observed across all treatments was the downregulation of trimethylamine N-oxide (TMAO) [Figure 3J].

Bile acid profiles, which are shaped by gut microbiota and influence intestinal homeostasis and neuromodulation^[50]^, were quantified in ileal content [Supplementary Figure 2B]. Bacterial interventions consistently drove a shift in BA composition toward a neuroprotective state. Specifically, they significantly reduced neurotoxic deoxycholic acid (DCA) and lithocholic acid (LCA) [Figure 3K-L], with DCA in the *B. wexlerae *MW-022 group being the sole exception. At the same time, they increased the neuroprotective bile acids ursodeoxycholic acid (UDCA) and tauroursodeoxycholic acid (TUDCA)^[51]^ [Figure 3M and N]. This rebalancing was reflected in the functional BA index, (UDCA + TUDCA)/(LCA + DCA). This metric was significantly elevated across all intervention groups, indicating a shift from toxic to a protective BA pool [Figure 3O, Supplementary Figure 2C and D]. This beneficial shift was further supported by marked reductions in the DCA/cholic acid (CA) and LCA/chenodeoxycholic acid (CDCA) ratios, both indicators of primary-to-secondary BA conversion, across all treated groups relative to the model [Figure 3P and Q, Supplementary Figure 2E and F]. Finally, all treatments also reversed the abnormal increase in histamine [Figure 3R].

### *Lachnospiraceae* strains reshape the gut microbiota structure in IBS mice

**Figure 4:**
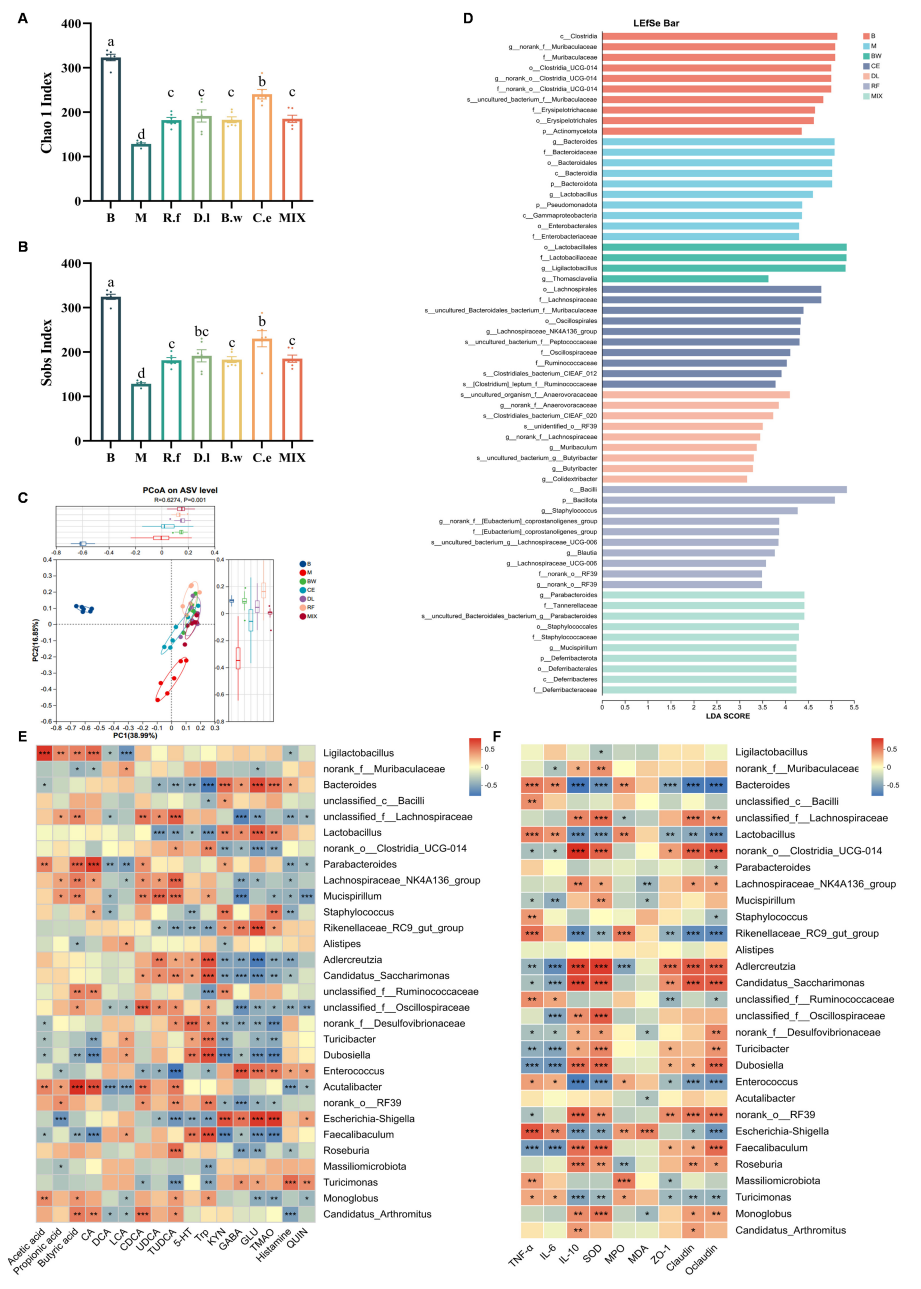
*Lachnospiraceae* strains restore gut microbiota structure in IBS mice (A and B) Alpha diversity indices: Chao1, and Sobs (C) Beta diversity: PCoA; (D) LEfSe analysis (LDA score > 2.0, *P* < 0.05); (E) Correlation heatmap between metabolites and gut microbes; (F) Correlation heatmap between disease phenotypic factors and gut microbes. (A and B) Data are presented as mean ± SEM. Different letters indicate statistically significant differences among groups (one-way ANOVA followed by Tukey HSD post hoc test, *P* < 0.05); (E and F) Spearman correlation analysis with Benjamini-Hochberg FDR correction. * adjusted *q* < 0.05, ** adjusted *q* < 0.01, *** adjusted *q* < 0.001; all displayed correlations also meet |*r*| > 0.6. TNF-α: Tumor necrosis factor-α; IL-6: interleukin-6; IL-10: interleukin-10; SOD: superoxide dismutase; MPO: myeloperoxidase; MDA: malondialdehyde; ZO-1: zonula occludens-1; IBS: irritable bowel syndrome; PCoA: principal coordinate analysis; LEfSe: linear discriminant analysis effect size; LDA: linear discriminant analysis; SEM: standard error of the mean; ANOVA: analysis of variance; HSD: honest significant difference; FDR: false discovery rate.

*Lachnospiraceae* interventions effectively reversed the disease-induced decline in gut microbiota α-diversity (Chao1 and Sobs indexes, Figure 4A and B) and regulated the overall microbial structure (PCoA, Figure 4C). Taxonomic analysis revealed that the IBS model group exhibited significant gut microbiota dysbiosis, characterized by a marked increase in Pseudomonadota and a significant decrease in Actinomycetota, alongside a notable reduction in beneficial genera such as *Adlercreutzia*, *Clostridia*_UCG-014, *Lachnospiraceae*_NK4A136_group, and *Roseburia* [Supplementary Figure 3].

LEfSe analysis further clarified potential microbial biomarkers specifically induced by individual strains [Figure 4D]: the *R.f* group enriched *Bacilli*, *Staphylococcus*, *Eubacterium_coprostanoligenes_group*, *Blautia*, *Lachnospiraceae*_UCG-006, and RF39. The *D.l* group specifically enriched *Anaerovoracaceae*, RF39, *Lachnospiraceae*, *Muribaculum*, *Butyribacter*, and *Colidextribacter*. The *B.w* group significantly enriched core members of the order *Lactobacillales*, including *Lactobacillaceae*, *Ligilactobacillus*, and *Thomasclavelia*. The *C.e* group showed enrichment focused on the *Lachnospirales *and *Oscillospirales *orders, including the key butyrate-producing genus *Lachnospiraceae_NK4A136_group*. The mix group primarily enriched *Parabacteroides*, *Staphylococcaceae*, *Mucispirillum*, and *Deferribacteraceae*.

To link these microbial shifts to functional outcomes, we performed correlation analyses between the microbiota, metabolites, and disease phenotypes. *Ligilactobacillus* and *Acutalibacter *positively correlated with all SCFAs. *Oscillospiraceae* was associated with the reversal of BA profiles (DCA, LCA, UDCA and TUDCA). In parallel, *Lachnospiraceae_NK4A136* correlated with improvements in LCA, UDCA and TUDCA. *Adlercreutzia*, *Candidatus_Saccharimonas*, *Oscillospiraceae*, *Desulfovibrionaceae*, *Dubosiella*, RF39, and *Faecalibaculum* showed positive correlations with the regulation of the GABA/Glu system. In contrast, *Monoglobus* and *Turicibacter* were positively linked exclusively to improvements of glutamine and TMAO [Figure 4E]. Finally, *Adlercreutzia* correlated with improvements in cytokines, oxidative-stress markers, and tight junction proteins; conversely, *Clostridia_UCG-014*, *Candidatus_Saccharimonas*, and *Dubosiella* correlated only with improvements in cytokines and tight junction proteins [Figure 4F].

### Potential activation of the gut-brain axis by *Lachnospiraceae* treatments

To investigate the gut-brain axis, we conducted multi-level correlation analyses to map the potential pathways of how *Lachnospiraceae* treatments alleviate IBS. A global analysis revealed significant associations among BAs, SCFAs, and neurological factors [Supplementary Figure 4A], establishing an across-system foundation.

**Figure 5:**
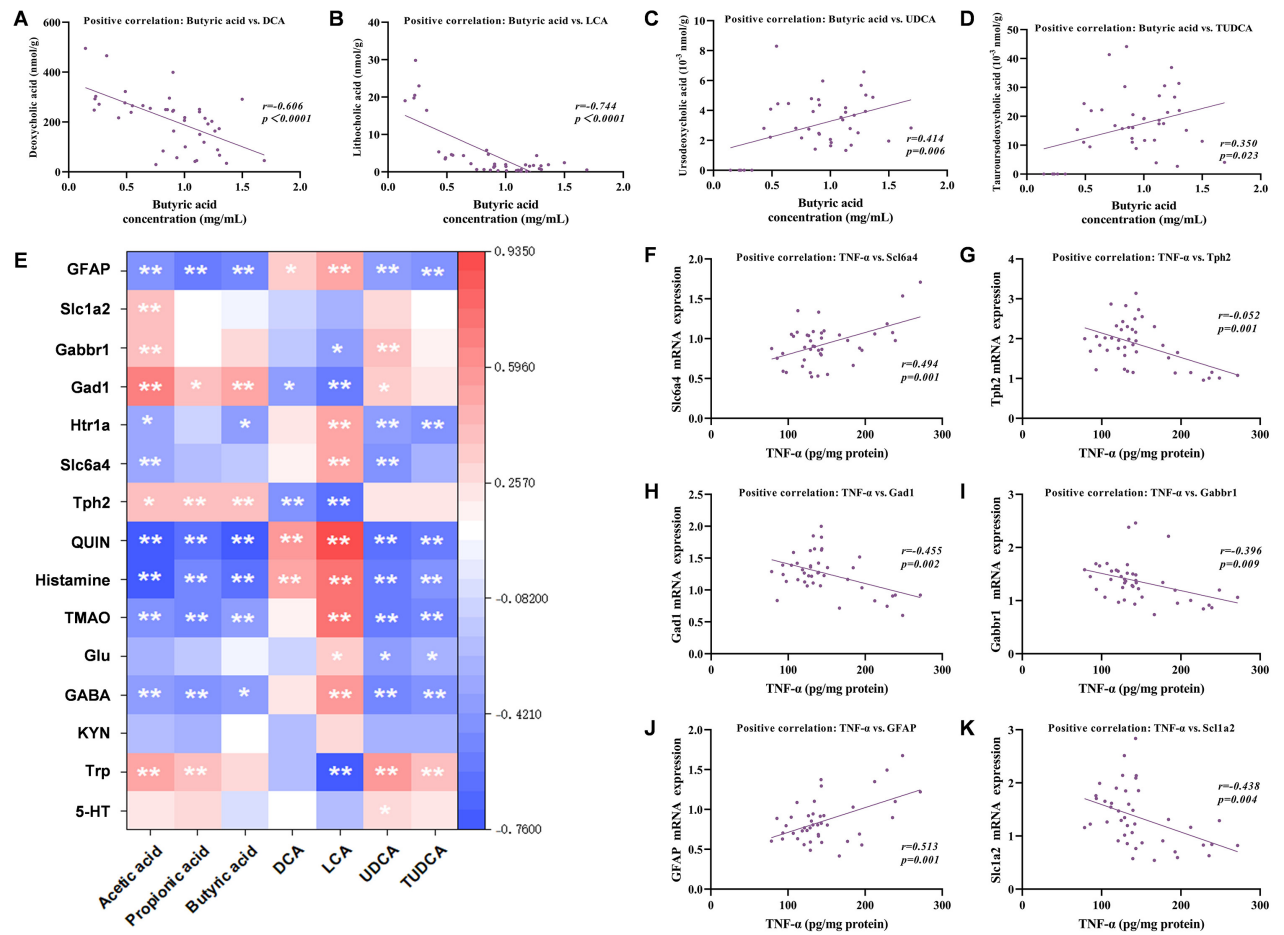
Potential activation of the gut-brain axis by *Lachnospiraceae* treatments. (A-D) Correlations between butyrate and BAs levels (DCA, LCA, UDCA, and TUDCA) (E); Heatmap of correlations between microbiota-derived metabolites with neurological factors; (F-K) Correlation of TNF-α with cerebral factors (*Tph2*, *Slc6a4*, *Gad1*, *Gabbr1*, GFAP, and *Slc1a2*). Note: (A-D, F-K) Spearman correlation analysis; *r* and *P* values are indicated in each panel. (E) Spearman correlation analysis with Benjamini-Hochberg FDR correction; *adjusted *q* < 0.05, **adjusted *q* < 0.01; all displayed correlations also meet |*r*| > 0.6. TMAO: trimethylamine N-oxide; GABA: γ-aminobutyric acid; KYN: kynurenine; Trp: tryptophan; 5-HT: serotonin; DCA: deoxycholic acid; LCA: lithocholic acid; UDCA: ursodeoxycholic acid; TUDCA: tauroursodeoxycholic acid; GFAP: glial fibrillary acidic protein; TNF-α: Tumor necrosis factor-α; BA: bile acids; FDR: false discovery rate.

Further dissection uncovered a pivotal regulatory cascade. Butyrate, a key SCFA produced by *Lachnospiraceae*, was significantly negatively correlated with toxic BAs (DCA and LCA, Figure 5A and B). It also showed positive correlations with neuroprotective BAs (UDCA and TUDCA, Figure 5C and D). Acetate showed similar trends [Supplementary Figure 4B-E], suggesting that its regulatory pathways concerning BAs may partially overlap with those of butyrate. This rebalancing profoundly influenced the nervous system [Figure 5E]. All three SCFAs positively correlated with *Gad1* and *Tph2*, but negatively correlated with GFAP, QUIN, histamine, TMAO, and GABA. Acetic acid was positively related to *Slc1a2*, *Gabbr1*, and *Trp*, and negatively related to *Htr1a* and *Slc6a4*. Conversely, propionic acid positively correlated with *Trp*, while butyric acid negatively correlated with *Htr1a*. Furthermore, shifts in BA profiles, specifically the downregulation of toxic BAs (DCA and LCA) and upregulation of neuroprotective BAs (UDCA and TUDCA), displayed close connections with *Gad1*, *Trp* and *Gabbr1*. Additionally, inflammatory cytokines (TNF-α and IL-6) demonstrated significant correlations with various neurological factors [Figure 5F-K, Supplementary Figure 4F-K]. This finding links peripheral inflammation to central outcomes.

## DISCUSSION

IBS is a highly prevalent functional gastrointestinal disorder. Due to the suboptimal efficacy of current pharmacotherapies, probiotics have emerged as promising low-risk alternatives^[52,53]^. Following our previous findings implicating the *Lachnospiraceae* family, we systematically evaluated the therapeutic potential of four representative strains. To eliminate the confounding effects of inter-individual microbiota variation, we standardized the gut microbiota baseline in IBS mice using SynCom-23. The constituent strains of SynCom-23 were largely absent from the LEfSe-identified biomarkers. This likely occurred because their baseline abundances were relatively uniform across all groups prior to the strain-specific interventions. Following the introduction of the specific *Lachnospiraceae* strains, some SynCom-23 members may have been competitively excluded or simply remained at their baseline levels. Consequently, the LEfSe analysis primarily captured a secondary ecological response: specifically, the expansion of indigenous, low-abundance bacteria that capitalized on the ecological disturbances induced by the interventions.

Our results showed that all *Lachnospiraceae* interventions effectively ameliorated core IBS phenotypes, including reducing weight loss, improving stool consistency, and alleviating visceral hypersensitivity. Furthermore, these interventions differentially corrected intestinal inflammation, oxidative stress, and barrier dysfunction, and crucially, improved behavioral abnormalities. These benefits were closely linked to the modulation of the gut microbiota structure and key metabolic pathways, particularly those involving BAs and SCFAs.

Delving into the strain-specific mechanisms revealed distinct therapeutic profiles. *B. wexlerae* MW-022 exhibited significant efficacy in suppressing intestinal inflammation and oxidative stress. While this strain influenced the expression of 5-HT, tryptophan, and kynurenine, its effects focused predominantly on the GABA system. *B. wexlerae* MW-022 potently upregulated the GABA-synthesizing rate-limiting enzyme *Gad1*, the glutamate transporter *Slc1a2*, and the GABA type B receptor *Gabbr1*. This suggests its ability to exert a multi-targeted influence on the GABAergic neurotransmission circuit, thereby enhancing GABA synthesis, accelerating glutamate clearance, and strengthening inhibitory signal reception. These coordinated actions collectively elevate central inhibitory tone^[54,55]^. *B. wexlerae* MW-022 prominently produces acetic acid, which also showed significant correlations with *Gad1*, *Slc1a2*, *Gabbr1*, GFAP, TMAO, and GABA. Additionally, it significantly enriched core members of *Ligilactobacillus* and *Thomasclavelia*. Through acetate production, *B. wexlerae* MW-022 may lower pH to inhibit pathogens and promote acidophilic lactobacilli that strengthen the barrier via bacteriocins^[56,57]^. *Thomasclavelia* converts primary to secondary BAs, which may ultimately affect gut-brain axis communication^[58]^.

*R. faecis *MW-024 exhibits notable efficacy in suppressing intestinal inflammation and oxidative stress, while prominently maintaining intestinal barrier integrity. Neurotransmitter analysis revealed that although this strain influences both 5-HT and GABA, its primary action centers on the GABA/Glu system. It effectively modulated the levels of GABA and TMAO, along with key factors such as *Gad1*, *Slc1a2* and the astrocytic marker GFAP, which plays a crucial supportive role in maintaining GABA/Glu balance^[54,55]^. Furthermore, the strain's primary metabolite, butyric acid, is known for its anti-inflammatory effects and role as a gut-brain axis modulator^[59]^, showing significant correlations with *Gad1*, GFAP, GABA and TMAO. Specifically, butyrate promotes the shift of the BAs pool toward a neuroprotective phenotype and, together with other SCFAs, regulates tryptophan metabolic flux. This favors the diversion of tryptophan into the serotonin synthesis pathway while maintaining the inhibitory tone of the GABAergic system. Notably, *R. faecis *MW-024 was enriched in *Eubacterium* *coprostanoligenes.* This bacterium not only helps maintain the intestinal immune barrier but also directly participates in the conversion of cholesterol to coprostanol, consuming a critical precursor for bile acid synthesis and thereby modulating bile acid metabolism^[60,61]^. This metabolic activity is also crucial for optimizing the microenvironment of GABAergic neurons. Additionally, the enriched taxa RF39 was also statistically correlated with GABA signalling.

*D. longicatena *MW-023 exerts broad and balanced regulatory effects on intestinal health. Although it successfully reversed the abnormal expression of GABA and glutamate in IBS mice, it did not significantly regulate most GABA/Glu enzymes. Instead, this strain upregulated the 5-HT synthesis rate-limiting enzyme Tph2 and the receptor Htr1a. Through these actions, it synergistically enhanced serotonergic neurotransmission at both the synthesis and receptor levels. Metabolically, *D. longicatena *MW-023 optimized the BAs pool and enriched *Anaerovoracaceae*, *Lachnospiraceae*, and *Butyribacter*-taxa involved in secondary BAs conversion, and SCFAs production^[56,62]^. Importantly, the gas-producing characteristic of *D. longicatena* may be detrimental to IBS patients with intestinal gas transport disorders. Although no obvious adverse effects were observed in this study, its safety margin still needs to be verified in a more sensitive abdominal distension model.

*C. eutactus *DSM107541 exhibited comparable anti-inflammatory and antioxidative capacities. Its mechanism focused on the “clearance–reception” axis of serotonergic signaling by targeting the reuptake transporter *Slc6a4* and the receptor *Htr1a*. Downregulation of *Slc6a4* may slow synaptic 5-HT reuptake, prolonging its action and synergizing with *Htr1a* to enhance the persistence and strength of 5-HT signaling. *D. longicatena* MW-023 and *C. eutactus* DSM107541 both modulate signal reception via *Htr1a*; however, they target distinct upstream processes. The former primarily promotes 5-HT biosynthesis, whereas the latter fine-tunes synaptic 5-HT homeostasis. This highlights how different strains achieve differentiated neuromodulation within the same system. Metabolically, *C. eutactus* DSM107541 produced all three SCFAs, predominantly butyric acid. Propionic and acetic acid correlated with the expression of *Trp*, while butyric and acetic acid were linked to *Htr1a*. These metabolic profiles confirm the strain's association with the 5-HT system. Finally, it enriched *Lachnospiraceae*, *Muribaculaceae*, *Oscillospiraceae*, *Ruminococcaceae*, *Peptococcaceae*, and *Lachnospiraceae_NK4A136_group*, indicating a selective promotion of a fiber-consuming, high SCFAs-yielding community^[63]^.

At the neuroactive molecular level, the strains exerted both shared and divergent effects. All four strains effectively downregulated GFAP to near-normal levels. In IBS, elevated GFAP reflects central neuroinflammation-driven astrocyte hyperplasia linked to visceral pain and gut dysfunction. Its restoration therefore suggests that this neuroinflammatory state is alleviated via the gut-brain axis, although confirmation at the protein and functional levels remains necessary^[64]^. Beyond this shared anti-inflammatory effect, GABAergic regulation revealed clear strain-specific divergence. Notably, all four strains synthesized GABA *in vitr*o; however, *in vivo*, only the *B. wexlerae* MW-022 group maintained GABA levels that were not significantly lower than those of the model group. We attribute this discrepancy to the fact that *in vitro* GABA production reflects inherent metabolic capacity.* In vivo*, GABA concentration results from a dynamic balance among microbial synthesis, host enzymatic degradation, and trans-epithelial transport^[65]^. Critically, *B. wexlerae* MW-022 was the only strain that simultaneously upregulated *Gad1*, *Slc1a2*, and *Gabbr1*. Through this coordinated enhancement of GABA synthesis, glutamate clearance, and inhibitory signal reception, it sustained a higher GABA steady-state level.

The other strains' incomplete GABA system regulation, in contrast, may be offset by host compensatory clearance, yielding a net reduction of intestinal GABA. While the strains diverged in their primary neurotransmitter targets - GABA/Glu versus 5-HT - histamine serves as a unifying link. Mast cell-derived histamine stimulates 5-HT release from enterochromaffin cells^[66]^, and central histaminergic neurons regulate GABA release via presynaptic H3 receptors^[67]^. In this study, all four strains effectively reversed the elevated ileal histamine levels in IBS mice. This common effect on histamine, together with the strains' differential modulation of 5-HT and GABA/Glu systems, suggests that *Lachnospiraceae* strains may suppress excessive mast cell activation to reduce histamine. In turn, this would facilitate synergy between these two neurotransmitter systems and contribute to the alleviation of IBS.

The mixed-strain intervention did not outperform individual strains in certain single metrics, such as *Htr1a* and GFAP, yet it excelled at reconstructing a complex, functionally complete gut ecosystem. We speculate that this phenomenon may stem from metabolic cross-interference and resource competition among multiple strains. Evidence indicates that interactions within multi-strain co-culture systems often follow a mixed “cooperation-antagonism” mode. Strains may achieve functional complementarity through metabolic exchange, yet competition for common substrates can redistribute metabolic fluxes, thereby reducing the net production of target metabolites. For instance, in one multi-strain probiotic co-culture system, enhanced metabolic exchange between strains coincided with a decrease in the net production of SCFAs and amino acids^[68,69]^. *In vitro* cultivation confirmed significant production of all three SCFAs. The microbiota of the MIX group was characterized by enriched *Lactobacillus* and *Lachnospiraceae_NK4A136_group*, plus a complex community comprising *Parabacteroides*, *Tannerellaceae*, *Staphylococcales*, *Mucispirillum*, and *Deferribacterota*. This diversified microbial structure indicates that the mixed intervention reshaped inter-species interaction networks and ecological niches, enhancing the regulation of intestinal homeostasis and providing a foundation for synergistic regulatory effects across pathways.

Post-intervention, *Adlercreutzia* showed close correlations with improvements in cytokines, oxidative-stress markers, tight-junction proteins, and GABA/Glu enzyme expressions, which was enriched in the R.f and C.e groups. While not a primary biomarker in this study, *Adlercreutzia* has been reported to act as a metabolic node linking SCFAs^[70]^ and secondary bile acids^[71]^. It may contribute to a gut microbial network^[72]^ that supports intestinal barrier integrity, immune homeostasis, and gut-brain signaling. Likewise, *Dubosiella* and *Candidatus Saccharimonas* were significantly related to improvements in cytokines, tight-junction proteins, and GABA/Glu enzyme expression. *Dubosiella* (especially *D. newyorkensis*) has been demonstrated as a SCFA-producing commensal bacterium with robust immunomodulatory and anti-inflammatory properties. It ameliorates colitis, hypertension, NAFLD, and aging-related pathologies through distinct but interconnected mechanisms. These include rebalancing Treg/Th17 responses via an L-lysine-AhR-IDO1-Kyn axis, and protecting the vascular endothelium by inhibiting pentosidine accumulation. Moreover, it consistently emerges as a beneficial responder in dietary and exercise interventions that improve metabolic and gut-brain axis health^[73-75]^. In humans, higher abundance of *Candidatus Saccharimonas* has been linked to better cognitive performance following a Mediterranean-style diet, suggesting a potential role in diet-gut-brain interactions^[76]^. These taxa may be promising targets in future studies.

This study has constructed a complete logical chain, from strain intervention through microbiota remodeling and metabolic changes, to neurotransmitter regulation to behavioral improvement. The inference at each stage does not rely solely on terminal correlation analysis, but is based on step-by-step verification. *In vitro* experiments confirmed each strain's metabolic capacity to produce known gut-brain axis signaling molecules such as butyric acid, acetic acid, and GABA. The *in vivo* changes of these metabolites align with their established biological functions, and the metabolite-neural associations exhibit a networked, multi-targeted pattern, reducing the likelihood of incidental findings. Nevertheless, we recognize that the gut-brain axis conclusions drawn here should be regarded as a mechanism hypothesis grounded in multi-dimensional evidence. Definitive causal validation will require future studies employing receptor knockout models, vagus nerve transection, or single-strain colonization in germ-free mice.

### Limitations

This study has several methodological limitations that warrant careful consideration. First, the colonization efficiency of individual SynCom-23 members was not monitored using qPCR or strain-specific primers. Additionally, no baseline microbiota sample was collected in the window between SynCom-23 colonization and the Lachnospiraceae intervention. Without direct colonization tracking and pre-intervention profiling, it is challenging to fully disentangle ecological remodeling driven by the target strains from the natural temporal drift of the synthetic community. Second, the inherent gas-producing property of *D. longicatena* may pose a risk for IBS patients with impaired intestinal gas handling. In addition, metabolic cross-interference among consortium members and the potential horizontal transfer of antibiotic resistance genes in the mixed-strain formulation should not be overlooked. Therefore, the long-term safety of these strains and their combination requires more comprehensive evaluation. Third, the gut-brain axis causal inferences drawn in this study rest primarily on correlation analyses and gene expression data. They lack direct functional validation through receptor-knockout models, pathway inhibition, or single-strain colonization experiments in germ-free mice. Consequently, the current conclusions should be regarded as a biologically plausible mechanistic hypothesis grounded in multi-dimensional evidence rather than definitive proof of causality. Future studies incorporating pre-intervention baseline sampling, strain-specific tracking of SynCom-23 members, and rigorous functional validation using gene-knockout or pathway-inhibition approaches will be essential to address these limitations. Such efforts will further consolidate the scientific foundation of this work.

### Conclusion

This study systematically reveals that the four representative strains effectively alleviate IBS symptoms in mice with potentially differential pathways: *B. wexlerae* MW-022 generates considerable acetic acid, stimulates *Lactobacillaceae* proliferation, and targets the GABA/Glu system. *R. faecis *MW-024 specializes in butyrate production and affects the GABA/Glu system. *D. longicatena *MW-023 enhances the 5-HT system at synthesis and reception levels, whereas *C. eutactus *DSM107541 modulates the 5-HT system via the “clearance-reception” mode. Furthermore, these strains in combination achieve more comprehensive effects on gut micro-ecology.

## DECLARATIONS

### Availability of data and materials

The original data supporting the findings presented in the article and Supplementary Materials will be made available from the corresponding author on request.

### Copyright

© The Author(s) 2026.

## References

[1] Camilleri M (2021). Diagnosis and treatment of irritable bowel syndrome: a review. JAMA..

[2] Staudacher HM, Mikocka-Walus A, Ford AC (2021). Common mental disorders in irritable bowel syndrome: pathophysiology, management, and considerations for future randomised controlled trials. Lancet Gastroenterol Hepatol..

[3] Simrén M, Barbara G, Flint HJ (2013). Intestinal microbiota in functional bowel disorders: a Rome foundation report. Gut..

[4] Linsalata M, Riezzo G, D’attoma B, Clemente C, Orlando A, Russo F (2018). Noninvasive biomarkers of gut barrier function identify two subtypes of patients suffering from diarrhoea predominant-IBS: a case-control study. BMC Gastroenterol..

[5] Drossman DA, Hasler WL (2016). Rome IV - Functional GI disorders: disorders of gut-brain interaction. Gastroenterology.

[6] Tanaka Y, Kanazawa M, Fukudo S, Drossman DA (2011). Biopsychosocial model of irritable bowel syndrome. J Neurogastroenterol Motil..

[7] Lacy BE, Mearin F, Chang L (2016). Bowel disorders. Gastroenterology..

[8] Mayer EA, Bradesi S, Chang L, Spiegel BMR, Bueller JA, Naliboff BD (2008). Functional GI disorders: from animal models to drug development. Gut..

[9] Sittipo P, Shim J, Lee Y (2019). Microbial metabolites determine host health and the status of some diseases. Int J Mol Sci..

[10] James SC, Fraser K, Young W, Mcnabb WC, Roy NC (2020). Gut microbial metabolites and biochemical pathways involved in irritable bowel syndrome: effects of diet and nutrition on the microbiome. J Nutr..

[11] Yang W, Tan H, Nie S (2025). A low-FODMAP diet enhances IBS symptom relief and gut microbiota homeostasis: a meta-analysis. Food Bioscience..

[12] Valdez-palomares F, Nambo-Venegas R, Uribe-García J (2021). Intestinal microbiota fingerprint in subjects with irritable bowel syndrome responders to a low FODMAP diet. Food Funct..

[13] Sciavilla P, Strati F, Di Paola M (2021). Gut microbiota profiles and characterization of cultivable fungal isolates in IBS patients. Appl Microbiol Biotechnol..

[14] Choi SI, Kim N, Nam RH (2023). The protective effect of *Roseburia faecis* against repeated water avoidance stress-induced irritable bowel syndrome in a wister rat model. J Cancer Prev..

[15] Pecyna P, Bykowska-Derda A, Gabryel M (2025). Blautia spp. in the gut microbiome: relation to dietary choices and to the nutritional status of patients with irritable bowel syndrome. Nutrition..

[16] Rui W, Li X, Wang L, Tang X, Yang J (2024). Potential applications of *Blautia wexlerae* in the regulation of host metabolism. Probiotics & Antimicro. Prot..

[17] Chen J, Zhao T, Li H (2024). Multi-omics analysis of gut microbiota and host transcriptomics reveal dysregulated immune response and metabolism in young adults with irritable bowel syndrome. Int J Mol Sci..

[18] Malinen E (2010). Association of symptoms with gastrointestinal microbiota in irritable bowel syndrome. World J Gastroenterol..

[19] Yang J, Wang P, Liu T (2021). Involvement of mucosal flora and enterochromaffin cells of the caecum and descending colon in diarrhoea-predominant irritable bowel syndrome. BMC Microbiol..

[20] Xu H, Ma C, Zhao F (2020). Adjunctive treatment with probiotics partially alleviates symptoms and reduces inflammation in patients with irritable bowel syndrome. Eur J Nutr..

[21] Taras D, Simmering R, Collins MD, Lawson PA, Blaut M (2002). Reclassification of Eubacterium formicigenerans Holdeman and Moore 1974 as Dorea formicigenerans gen. nov., comb. nov., and description of Dorea longicatena sp. nov., isolated from human faeces. Int J Syst Evol Microbiol..

[22] Duncan SH (2002). Growth requirements and fermentation products of Fusobacterium prausnitzii, and a proposal to reclassify it as Faecalibacterium prausnitzii gen. nov., comb. nov. Int J Syst Evol Microbiol..

[23] Simon DW, Rogers MB, Gao Y (2020). Depletion of gut microbiota is associated with improved neurologic outcome following traumatic brain injury. Brain Res..

[24] Bharwani A, Mian MF, Foster JA, Surette MG, Bienenstock J, Forsythe P (2016). Structural & functional consequences of chronic psychosocial stress on the microbiome & host. Psychoneuroendocrinology..

[25] Zhang J, Wang X, Lu Y, Qiao Y, Xia X, Tu J (2010). Microstructure and infrared reflectance modulation properties in DC-sputtered tungsten oxide films. J Solid State Electrochem..

[26] Chen C, Lu M, Pan Q (2015). Berberine improves intestinal motility and visceral pain in the mouse models mimicking diarrhea-predominant irritable bowel syndrome (IBS-D) symptoms in an opioid-receptor dependent manner. PLoS ONE..

[27] Furuichi M, Kawaguchi T, Pust M (2024). Commensal consortia decolonize Enterobacteriaceae via ecological control. Nature..

[28] Weiss AS, Burrichter AG, Durai Raj AC (2022). *In vitro* interaction network of a synthetic gut bacterial community. The ISME Journal..

[29] Weiss AS, Niedermeier LS, Von Strempel A (2023). Nutritional and host environments determine community ecology and keystone species in a synthetic gut bacterial community. Nat Commun..

[30] Yim SK, Kim SW, Lee ST (2021). Efficient stool collection methods for evaluating the diarrhea score in mouse diarrhea models. In Vivo..

[31] Dellu F, Mayo W, Cherkaoui J, Le Moal M, Simon H (1992). A two-trial memory task with automated recording: study in young and aged rats. Brain Res..

[32] Seibenhener ML, Wooten MC (2015). Use of the open field maze to measure locomotor and anxiety-like behavior in mice. J Vis Exp..

[33] Zhang S, Nie Q, Sun Y (2024). Bacteroides uniformis degrades β-glucan to promote Lactobacillus johnsonii improving indole-3-lactic acid levels in alleviating colitis. Microbiome..

[34] Liu C, Zhao D, Ma W (2015). Denitrifying sulfide removal process on high-salinity wastewaters in the presence of Halomonas sp. Appl Microbiol Biotechnol..

[35] Chen S, Zhou Y, Chen Y, Gu J (2018). fastp: an ultra-fast all-in-one FASTQ preprocessor. Bioinformatics..

[36] Magoč T, Salzberg SL (2011). FLASH: fast length adjustment of short reads to improve genome assemblies. Bioinformatics..

[37] Edgar RC (2013). UPARSE: highly accurate OTU sequences from microbial amplicon reads. Nat Methods..

[38] Wang Q, Garrity GM, Tiedje JM, Cole JR (2007). Naïve Bayesian classifier for rapid assignment of rRNA sequences into the new bacterial taxonomy. Appl Environ Microbiol..

[39] Schloss PD, Westcott SL, Ryabin T (2009). Introducing mothur: Open-source, platform-independent, community-supported software for describing and comparing microbial communities. Appl Environ Microbiol..

[40] Segata N, Izard J, Waldron L (2011). Metagenomic biomarker discovery and explanation. Genome Biol..

[41] Barberán A, Bates ST, Casamayor EO, Fierer N (2012). Using network analysis to explore co-occurrence patterns in soil microbial communities. The ISME Journal..

[42] Zhang S, Sun Y, Li Y, Chen C, Nie S (2026). A synbiotic combination of arabinogalactan and Bacteroides thetaiotaomicron ameliorates ulcerative colitis in mice. Food Res Int..

[43] Li S, Tan H, Yang J (2025). Effects of three homogalacturonan-type pectins on mice with metabolic syndrome. J Agric Food Chem..

[44] Hao M, Song J, Zhai X (2023). Improvement of loperamide-hydrochloride-induced intestinal motility disturbance by Platycodon grandiflorum polysaccharides through effects on gut microbes and colonic serotonin. Front. Cell. Infect. Microbiol..

[45] Zhao L, Zhang F, Ding X (2018). Gut bacteria selectively promoted by dietary fibers alleviate type 2 diabetes. Science..

[46] Hosseinkhani F, Dubbelman A, Karu N, Harms AC, Hankemeier T (2021). Towards standards for human fecal sample preparation in targeted and untargeted LC-HRMS studies. Metabolites..

[47] Luczynski P, Mcvey Neufeld K, Oriach CS, Clarke G, Dinan TG, Cryan JF (2016). Growing up in a bubble: using germ-free animals to assess the influence of the gut microbiota on brain and behavior. Int J Neuropsychopharmacol..

[48] O’Mahony S, Clarke G, Borre Y, Dinan T, Cryan J (2015). Serotonin, tryptophan metabolism and the brain-gut-microbiome axis. Behav Brain Res..

[49] Pokusaeva K, Johnson C, Luk B (2016). GABA‐producing *Bifidobacterium dentium* modulates visceral sensitivity in the intestine. Neurogastroenterology Motil..

[50] Wahlström A, Sayin SI, Marschall H, Bäckhed F (2016). Intestinal crosstalk between bile acids and microbiota and its impact on host metabolism. Cell Metab..

[51] Ackerman HD, Gerhard GS (2016). Bile acids in neurodegenerative disorders. Front. Aging Neurosci..

[52] Sperber AD, Bangdiwala SI, Drossman DA (2021). Worldwide prevalence and burden of functional gastrointestinal disorders, results of Rome foundation global study. Gastroenterology..

[53] Black CJ, Burr NE, Camilleri M (2020). Efficacy of pharmacological therapies in patients with IBS with diarrhoea or mixed stool pattern: systematic review and network meta-analysis. Gut..

[54] Zhang B, Vogelzang A, Miyajima M (2021). B cell-derived GABA elicits IL-10+ macrophages to limit anti-tumour immunity. Nature..

[55] Jiang L, Zhu B, Zhao Y (2019). Membralin deficiency dysregulates astrocytic glutamate homeostasis, leading to ALS-like impairment. J Clin Investig..

[56] Macfarlane GT, Macfarlane S (2012). Bacteria, colonic fermentation, and gastrointestinal health. J AOAC Int..

[57] Cotter PD, Ross RP, Hill C Bacteriocins - a viable alternative to antibiotics? *Nat Rev Microbiol.* 2012;11:95-105.

[58] Sato Y, Atarashi K, Plichta DR (2021). Novel bile acid biosynthetic pathways are enriched in the microbiome of centenarians. Nature..

[59] Canani RB, Di Costanzo M, Leone L, Pedata M, Meli R (2011). Potential beneficial effects of butyrate in intestinal and extraintestinal diseases. World J Gastroenterol..

[60] Kenny DJ, Plichta DR, Shungin D (2020). Cholesterol metabolism by uncultured human gut bacteria influences host cholesterol level. Cell Host Microbe..

[61] Bai D, Zhao J, Wang R (2024). Eubacterium coprostanoligenes alleviates chemotherapy-induced intestinal mucositis by enhancing intestinal mucus barrier. Acta Pharmaceutica Sinica B..

[62] Winston JA, Theriot CM (2019). Diversification of host bile acids by members of the gut microbiota. Gut Microbes..

[63] Louis P, Flint HJ (2016). Formation of propionate and butyrate by the human colonic microbiota. Environ Microbiol..

[64] Li W, Zhang L, Chen S (2025). Spinal astrocytes in chronic visceral pain. Dev Neurobiol..

[65] Ikegami M, Narabayashi H, Nakata K (2024). Intervention in gut microbiota increases intestinal γ-aminobutyric acid and alleviates anxiety behavior: a possible mechanism via the action on intestinal epithelial cells. Front. Cell. Infect. Microbiol..

[66] Wood JD (2020). Serotonergic integration in the intestinal mucosa. Curr Pharm Des..

[67] Schlicker E, Kathmann M

[68] Zampieri G, Efthimiou G, Angione C (2023). Multi-dimensional experimental and computational exploration of metabolism pinpoints complex probiotic interactions. Metab Eng..

[69] Fredua-agyeman M, Stapleton P, Basit AW, Gaisford S (2017). Microcalorimetric evaluation of a multi-strain probiotic: Interspecies inhibition between probiotic strains. J Funct Foods..

[70] Shin AS, Xing Y, Waseem MR (2025). Microbiota and short chain fatty acid relationships underlie clinical heterogeneity and identify key microbial targets in irritable bowel syndrome (IBS). Sci Rep..

[71] Wylensek D, Hitch TCA, Riedel T (2020). A collection of bacterial isolates from the pig intestine reveals functional and taxonomic diversity. Nat Commun..

[72] Zhang Z, Yang Z, Lin S (2025). Probiotic-induced enrichment of *Adlercreutzia equolifaciens* increases gut microbiome wellness index and maps to lower host blood glucose levels. Gut Microbes..

[73] Zhang Y, Tu S, Ji X (2024). Dubosiella newyorkensis modulates immune tolerance in colitis via the L-lysine-activated AhR-IDO1-Kyn pathway. Nat Commun..

[74] Liu T, Chen M, Zhang C (2025). Hypertension inhibition by Dubosiella newyorkensis via reducing pentosidine synthesis. npj Biofilms Microbiomes..

[75] Ye X, Sun P, Lao S (2023). Fgf21-Dubosiella axis mediates the protective effects of exercise against NAFLD development. Life Sci..

[76] Solch R, Engler-chiurazzi E, Harper C (2022). A mediterranean diet enhances cognitive function and modulates the gut microbiota. Curr Dev Nutr..

