# Supplementary material for: Therapeutic potential of Lachnospiraceae strains in irritable bowel syndrome via differential gut-brain pathways
Source: Microbiome Res Rep. 2026 May 29;5(2):12. doi: 10.20517/mrr.2026.08 (PMC13246493; doi:10.20517/mrr.2026.08)
Supplement: Supplementary file 1 [file mrr-5-2-12-SupplementaryMaterials.pdf]

Supplementary Materials

Therapeutic potential of *Lachnospiraceae* strains in irritable bowel syndrome via differential gut-brain pathways

Wanyu Yang, Shuigen Bian, Huizi Tan

State Key Laboratory of Food Science and Resources, China-Canada Joint Laboratory of Food Science and Technology (Nanchang), Nanchang University, Nanchang 330047, Jiangxi, China.

Correspondence to: Dr. Huizi Tan, State Key Laboratory of Food Science and Resources, China-Canada Joint Laboratory of Food Science and Technology (Nanchang), Nanchang University, Nanchang 330047, Jiangxi, China. E-mail: huizi.tan@ncu.edu.cn

ORCID: Huizi Tan (0000-0001-9674-7835)

The Supplementary materials includes:

Supplementary Table 1. Composition of the synthetic bacterial community (SynCom-23).

Supplementary Table 2. Primer sequences used for RT-qPCR.

Supplementary Figure 1. H&E staining of colon section (scale bar = 400 μm).

Supplementary Figure 2. GABA production by *Lachnospiraceae* strains and ileal BAs levels in IBS mice. (A) GABA produced by the four *Lachnospiraceae* strains and their mixture. (B) Total bile acids (Total BAs). (C) Toxic bile acids: deoxycholic acid (DCA) and lithocholic acid (LCA). (D) Total protective bile acids: ursodeoxycholic acid (UDCA) and tauroursodeoxycholic acid (TUDCA). (E) Cholic acid (CA). (F) Chenodeoxycholic acid (CDCA).Note: Different letters indicate statistical difference among groups. Note: Data are presented as mean ± SEM. Different letters indicate statistically significant differences among groups (one-way ANOVA followed by

Tukey HSD post hoc test, $p < 0.05$).

Supplementary Figure 3. Taxonomic composition of gut microbiota in IBS mice treated with *Lachnospiraceae* strains. (A) Phylum-level bar plot. (B) Genus-level bar plot. (C) Relative abundance of Pseudomonadota and Actinomycetota. (D) Relative abundance of *Clostridia*_UCG-014, *Muribaculaceae, Adlercreutzia,* and *Roseburia*. Note: Data are presented as mean ± SEM. Different letters indicate statistically significant differences among groups (one-way ANOVA followed by Tukey HSD post hoc test, $p < 0.05$).

Supplementary Figure 4. Multi-level correlation analysis of gut-brain axis. (A) Correlation heatmap. (B-E).Correlations between acetic acid and BAs levels (DCA, LCA, UDCA, and TUDCA). (F-K) Correlations of IL-6 with cerebral gene expression (*Tph2*, *Slc6a4*, *Gad1*, *Gabbr1*, GFAP, and *Slc1a2*). $p < 0.05$ was considered statistically significant. Note: (A) Spearman correlation analysis with Benjamini-Hochberg FDR correction; * indicates adjusted $q < 0.05$, $|r| > 0.6$. (B-E) Spearman correlation analysis; r and p values are indicated in each panel.

Supplementary Table 1. Composition of the synthetic bacterial community (SynCom-23)

**Synthetic Bacterial Community (SynCom-23)[a]**			
**Strain**	**concentration**	**Strain**	**concentration**
Bacteroides stercoris MW-001	10^8 CFU/mL	*Bifidobacterium longum* MW-013	10^8 CFU/mL
Bacteroides vulgatus MW-002	10^8 CFU/mL	*Collinsella aerofaciens* MW-014	10^8 CFU/mL
Bacteroides caccae MW-003	10^8 CFU/mL	*Streptococcus luteiensis* MW-015	0.5×10^8 CFU/mL
Bacteroides xylanisolvens MW-004	10^8 CFU/mL	*Streptococcus salivarius* MW-016	0.5×10^8 CFU/mL
Bacteroides uniformis MW-005	10^8 CFU/mL	*Escherichia coli* MW-017	10^8 CFU/mL
Bacteroides	10^8 CFU/mL	*Ruminococcus*	10^8 CFU/mL

thetaiotaomicron MW-006		*bromii* MW-018	
Bacteroides cellulosilyticus MW-007	10^8 CFU/mL	*Prevotella ihumii* MW-019	0.5×10^8 CFU/mL
Bacteroides dorei MW-008	10^8 CFU/mL	*Prevotella jejuni* MW-020	0.5×10^8 CFU/mL
Bacteroides ovatus MW-009	10^8 CFU/mL	*Phocaeicola dore* MW-021*i*	10^8 CFU/mL
Akkermansia muciniphila MW-010	10^8 CFU/mL	*Eubacterium Siraeum* DSM 15702	10^8 CFU/mL
paraclostridium benzoelyticum MW-011	10^8 CFU/mL	*Clostridium Leptum* DSM 753	10^8 CFU/mL
Agathobacter rectalis MW-012	10^8 CFU/mL		

[a] All strains were isolated in-house from healthy human feces, except for *E. siraeum* DSM 15702 and *C. leptum* DSM 753, which were obtained from DSMZ, Germany.

Supplementary Table 2. Primer sequences used for RT-qPCR

Target genes	Primer sequences	References
CGRP	F: GAAGAAGAAGTTCGCCTGCT<br>R: GATTCCCACACCGCTTAGAT	[1]
TRPV1	F: CGAGGATGGGAAGAATAACTCACTG<br>P: GGATGATGAAGACAGCCTTGAAGTC	[2]
ZO-1	F: AGGACACCAAAGCATGTGAG<br>R: GGCATTCCTGCTGGTTACA	[3]
Occludin	F: TTGAAAGTCCACCTCCTTACAGA<br>R: CCGGATAAAAAGAGTACGCTGG	[3]
Claudin1	F: CCTGCCCCAGTGGAAGATTT<br>R: AAACGCAGGACATCCACAGT	[3]
Htr1a	F: ACAGGGCGGTGGGGACTC<br>R: CAAGCAGGCGGGGACATAGG	[4]
Tph2	F: TCCTTTGACCCAAAGACGAC<br>R: TTCAATGCTCTGCGTGTAGG	[5]
Slc6a4	F: GGCTGAGATGAGGAACGAAG<br>R: CTATCCAAACCCAGCGTGAT	[5]

Gad1	F: AGGCAGTCCTCCAAGAACCT<br>R: CCGTTCTTAGCTGGAAGCAG	[6]
Gabbr1	F: GA GGACGTGAATAGCCGCAG<br>R: CTGGATCACACTTGCTGTCGT	[7]
Slc1a2	F: GGCAGCTGGGGATGTACA<br>R: ACGCTGGGGAGTTTATTCAAGAAT	[8]
GFAP	F: CCTTCTGACACGGATTTGGT<br>R: TAAGCTAGCCCTGGACATCG	[9]
β-actin	F: GGCTGTATTCCCCTCCATCG<br>R: CCAGTTGGTAACAATGCCATGT	[10]

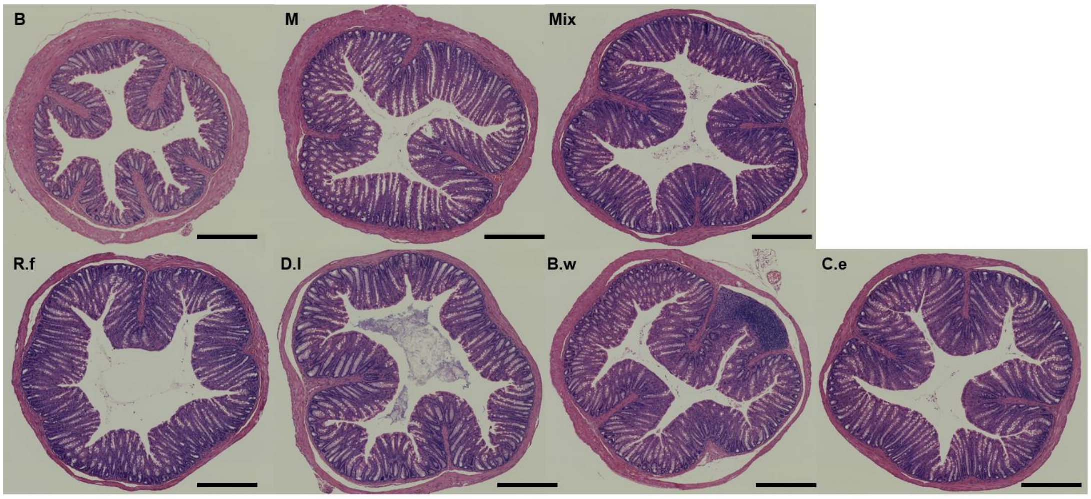

Supplementary Figure 1. H&E staining of colon sections (scale bar = 400 μm).

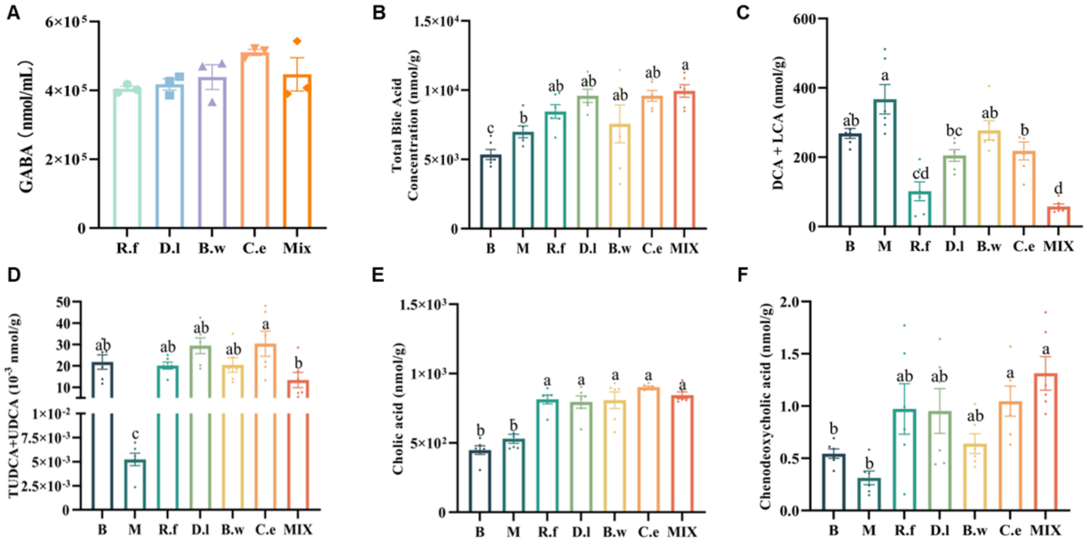

Supplementary Figure 2. GABA production by *Lachnospiraceae* strains and ileal BAs levels in IBS mice. (A) GABA produced by the four *Lachnospiraceae* strains and

their mixture. (B) Total bile acids (Total BAs). (C) Toxic bile acids: deoxycholic acid (DCA) and lithocholic acid (LCA). (D) Total protective bile acids: ursodeoxycholic acid (UDCA) and tauroursodeoxycholic acid (TUDCA). (E) Cholic acid (CA). (F) Chenodeoxycholic acid (CDCA). Note: Data are presented as mean ± SEM. Different letters indicate statistically significant differences among groups (one-way ANOVA followed by Tukey HSD post hoc test, $p < 0.05$).

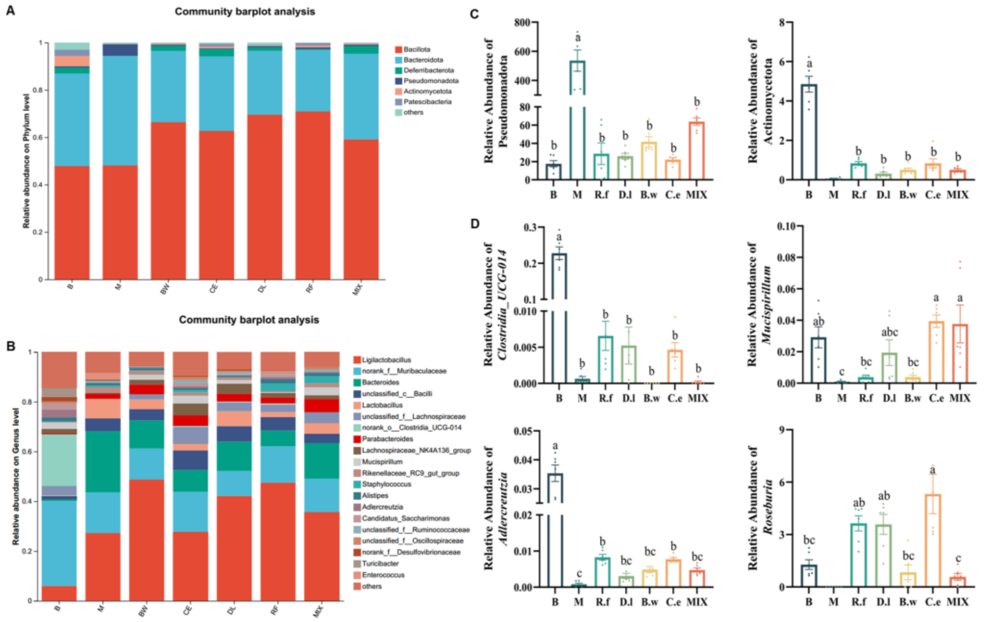

Supplementary Figure 3. Taxonomic composition of gut microbiota in IBS mice treated with *Lachnospiraceae* strains. (A) Phylum-level bar plot. (B) Genus-level bar plot. (C) Relative abundance of Pseudomonadota and Actinomycetota. (D) Relative abundance of *Clostridia*_UCG-014, *Muribaculaceae, Adlercreutzia,* and *Roseburia*. Note: Data are presented as mean ± SEM. Different letters indicate statistically significant differences among groups (one-way ANOVA followed by Tukey HSD post hoc test, $p < 0.05$).

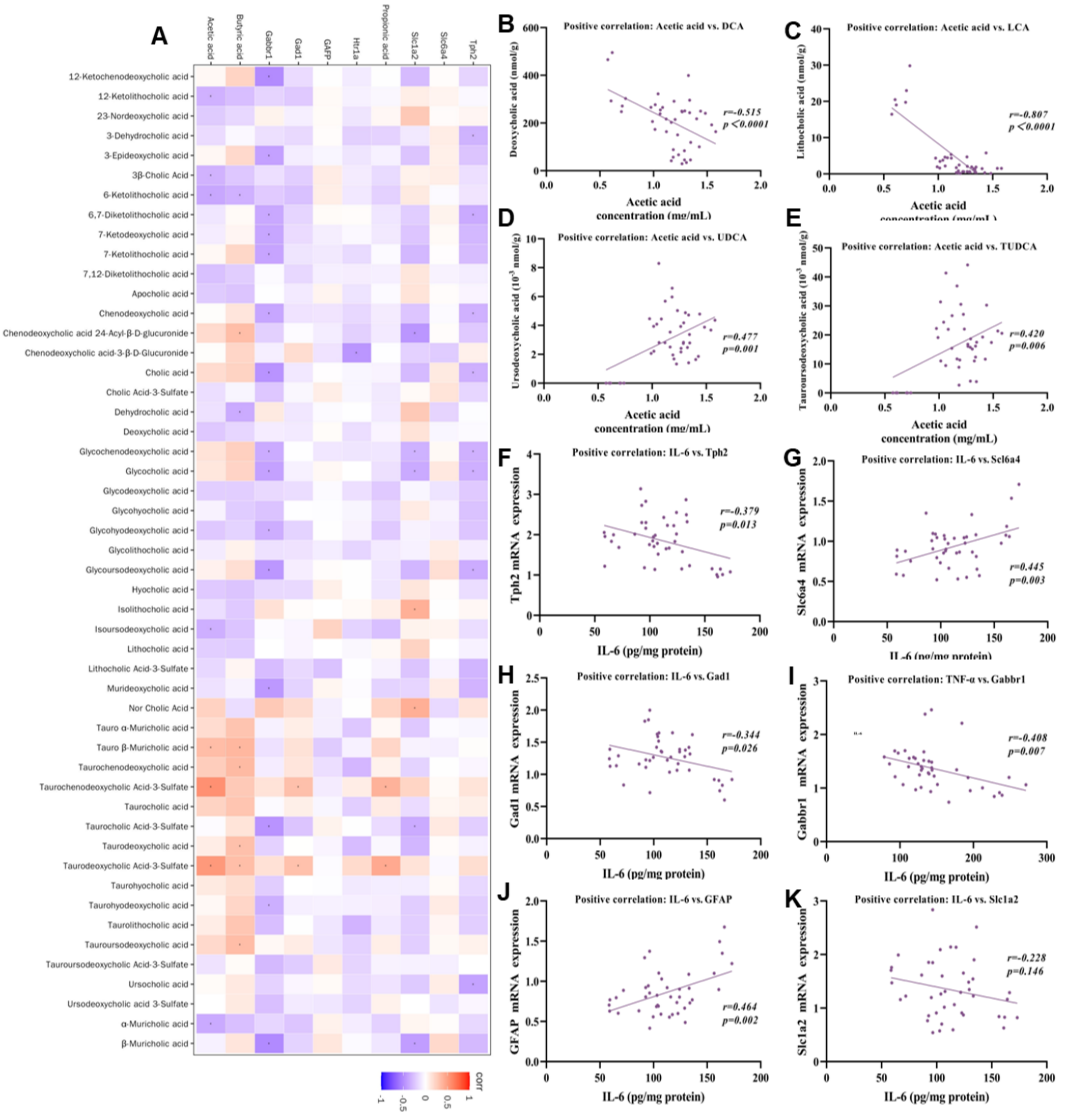

Supplementary Figure 4. Multi-level correlation analysis of gut-brain axis. (A) Correlation heatmap. (B-E).Correlations between acetic acid and BAs levels (DCA, LCA, UDCA, and TUDCA). (F-K) Correlations of IL-6 with cerebral gene expression (*Tph2*, *Slc6a4*, *Gad1*, *Gabbr1*, GFAP, and *Slc1a2*). $p < 0.05$ was considered statistically significant. Note: (A) Spearman correlation analysis with Benjamini-Hochberg FDR correction; * indicates adjusted $q < 0.05$, $|r| > 0.6$. (B-E) Spearman correlation analysis; r and p values are indicated in each panel.

REFERENCES

[1] Minamiyama M, Katsuno M, Adachi H, Doi H, Kondo N, et al. Naratriptan mitigates CGRP1-associated motor neuron degeneration caused by an expanded polyglutamine repeat tract. *Nat Med*. 2012;18:1531-38. DOI: 10.1038/nm.2932

[2] Zhang Y, Hou B, Liang P, Lu X, Wu Y, et al. TRPV1 channel mediates NLRP3

inflammasome-dependent neuroinflammation in microglia. *Cell Death Dis*. 2021;12:1159. DOI: 10.1038/s41419-021-04450-9

[3] Nie X, Li Q, Ji H, Zhang S, Wang Y, et al. *Bifidobacterium longum* NSP001-derived extracellular vesicles ameliorate ulcerative colitis by modulating T cell responses in gut microbiota-(in)dependent manners. *npj Biofilms Microbiomes*. 2025;11:27. DOI: 10.1038/s41522-025-00663-4

[4] Bianchi P, Ciani E, Guidi S, Trazzi S, Felice D, et al. Early pharmacotherapy restores neurogenesis and cognitive performance in the Ts65Dn mouse model for Down syndrome. *J Neurosci*. 2010;30:8769-79. DOI: 10.1523/JNEUROSCI.0534-10.2010

[5] Shioda N, Imai Y, Yabuki Y, Sugimoto W, Yamaguchi K, et al. Dopamine D2L receptor deficiency causes stress vulnerability through 5-HT1A receptor dysfunction in serotonergic neurons. *J Neurosci*. 2019;39:7551-63. DOI: 10.1523/JNEUROSCI.0079-19.2019

[6] Zhang B, Vogelzang A, Miyajima M, Sugiura Y, Wu Y, et al. B cell-derived GABA elicits IL-10^{+} macrophages to limit anti-tumour immunity. *Nature*. 2021;599:471-6. DOI: 10.1038/s41586-021-04082-1

[7] Longqiu Y, Pengcheng L, Xuejie F, Peng Z. A miRNAs panel promotes the proliferation and invasion of colorectal cancer cells by targeting GABBR1. *Cancer Med*. 2016;5:2022-31. DOI: 10.1002/cam4.760

[8] Jiang LL, Zhu B, Zhao Y, Li X, Liu T, et al. Membralin deficiency dysregulates astrocytic glutamate homeostasis, leading to ALS-like impairment. *J Clin Invest*. 2019;129:3103-20. DOI: 10.1172/JCI127695

[9] Bussian TJ, Aziz A, Meyer CF, Swenson BL, van Deursen JM, et al. Clearance of senescent glial cells prevents tau-dependent pathology and cognitive decline. *Nature*. 2018;562:578-82. DOI: 10.1038/s41586-018-0543-y

[10] Zhang S, Sun Y, Gao L, Zhang Z, Yu Y, et al. Synergistic protective effects of β-glucan and *Lactobacillus johnsonii* NSP009 in ulcerative colitis. *Food Funct*. 2025;16:8345-58. DOI: 10.1039/d5fo02391b
